# Tbx2 Controls Lung Growth by Direct Repression of the Cell Cycle Inhibitor Genes *Cdkn1a* and *Cdkn1b*

**DOI:** 10.1371/journal.pgen.1003189

**Published:** 2013-01-17

**Authors:** Timo H-W. Lüdtke, Henner F. Farin, Carsten Rudat, Karin Schuster-Gossler, Marianne Petry, Phil Barnett, Vincent M. Christoffels, Andreas Kispert

**Affiliations:** 1 Institute for Molecular Biology, Medizinische Hochschule Hannover, Hannover, Germany; 2 Department of Anatomy, Embryology and Physiology, Heart Failure Research Center, Academic Medical Center, University of Amsterdam, Amsterdam, The Netherlands; Columbia University Medical Center, United States of America

## Abstract

Vertebrate organ development relies on the precise spatiotemporal orchestration of proliferation rates and differentiation patterns in adjacent tissue compartments. The underlying integration of patterning and cell cycle control during organogenesis is insufficiently understood. Here, we have investigated the function of the patterning T-box transcription factor gene *Tbx2* in lung development. We show that lungs of *Tbx2*-deficient mice are markedly hypoplastic and exhibit reduced branching morphogenesis. Mesenchymal proliferation was severely decreased, while mesenchymal differentiation into fibrocytes was prematurely induced. In the epithelial compartment, proliferation was reduced and differentiation of alveolar epithelial cells type 1 was compromised. Prior to the observed cellular changes, canonical Wnt signaling was downregulated, and *Cdkn1a* (*p21*) and *Cdkn1b* (*p27*) (two members of the Cip/Kip family of cell cycle inhibitors) were strongly induced in the *Tbx2*-deficient lung mesenchyme. Deletion of both *Cdkn1a* and *Cdkn1b* rescued, to a large degree, the growth deficits of *Tbx2*-deficient lungs. Prolongation of *Tbx2* expression into adulthood led to hyperproliferation and maintenance of mesenchymal progenitor cells, with branching morphogenesis remaining unaffected. Expression of Cdkn1a and Cdkn1b was ablated from the lung mesenchyme in this gain-of-function setting. We further show by ChIP experiments that Tbx2 directly binds to *Cdkn1a* and *Cdkn1b* loci *in vivo*, defining these two genes as direct targets of Tbx2 repressive activity in the lung mesenchyme. We conclude that Tbx2-mediated regulation of *Cdkn1a* and *Cdkn1b* represents a crucial node in the network integrating patterning information and cell cycle regulation that underlies growth, differentiation, and branching morphogenesis of this organ.

## Author Summary

During organ formation, proliferation rates and differentiation patterns vary widely between different stages and tissue compartments. It is poorly understood how cell cycle progression is locally controlled and integrated with patterning processes in these developmental programs. Here, we used the mouse lung as a model to study how growth and differentiation are controlled on a transcriptional level. Combining genetic loss- and gain-of-function approaches, we show that the T-box transcription factor gene *Tbx2* is required and sufficient to direct appropriate lung growth by maintaining proliferation and inhibiting differentiation in the mesenchymal compartment of the lung. We found that expression of the cell cycle inhibitor genes *Cdkn1a* (*p21*) and *Cdkn1b* (*p27*) inversely correlates with expression of Tbx2 and that deletion of both genes rescues, to a large degree, the growth deficits of *Tbx2*-mutant lungs. We further show by biochemical assays that Tbx2 directly binds to *Cdkn1a* and *Cdkn1b* loci *in vivo*, defining these two genes as direct targets of Tbx2 repressive activity in the lung mesenchyme. We conclude that Tbx2-mediated regulation of *Cdkn1a* and *Cdkn1b* represents a crucial module for the tissue-specific control of cell cycle progression that underlies growth, differentiation, and branching morphogenesis of this organ.

## Introduction

The development of organs and organisms depends on the precise control of the progression through and the exit from the cell cycle to achieve appropriate patterns of proliferation and differentiation in time and space. Progression through the cell cycle is regulated predominantly by a series of serine/threonine kinases, the cyclin-dependent kinases (CDKs) that link proliferative signals with mechanical aspects of cell duplication. CDK function is controlled by a variety of mechanisms, including a group of molecules that inhibits CDK activity by complex formation. These CDK inhibitors (CKIs) have been categorized into two families, the Cip/Kip (Cdkn1) family with three members in mammals (Cdkn1a, Cdkn1b, Cdkn1c (also known as p21, p27 and p57)), that inhibit all kinases involved in G_1_/S transition, and the Ink4 (Cdkn2) family with four mammalian members (Cdkn2a, Cdkn2b, Cdkn2c, Cdkn2d (also known as p16/p19ARF, p15, p18, p19)) that specifically inhibit Cdk4 and Cdk6. Biochemical and cell culture experiments have identified CKIs as primary effectors of signaling pathways that control cell cycle exit, an event critical for differentiation. Expression or stability of CKIs is reduced in tumors, and deletion of six of the seven family members leads to organ hyperplasia and increased tumor susceptibility. In contrast to the obvious relevance of CKIs in tissue homeostasis, their role in development of tissues and organs, and the transcriptional mechanisms that mediate their precise temporal and spatial expression in the embryo have been much less well defined. This may relate to functional redundancy between family members as well as to the complexity of their regulatory modules (for reviews on CKIs see [1]–[3]).

T-box (*Tbx*) genes encode an evolutionary conserved family of transcription factors that regulate patterning and differentiation processes during vertebrate development [4]. *Tbx2* and *Tbx3* are two closely related members of the Tbx2-subfamily that are required in the development of numerous organs during mammalian embryogenesis including the heart, the palate, the limbs, and the liver [5]–[10]. In these contexts, these two transcriptional repressors mainly seem to regulate cell fate decisions and differentiation. However, *in vitro* studies indicated a role for Tbx2 and Tbx3 in the progression of the cell cycle [11]–[13]. Expression of *Tbx2* and *Tbx3* is upregulated in a number of tumors including those of the breast, pancreas, liver and bladder, and in melanomas, and both genes can function as immortalizing agents to bypass senescence, i.e. escape irreversible growth arrest (for reviews see [14], [15]). In cell culture assays, this phenomenon is mediated by transcriptional repression of *Cdkn1a* and *Cdkn2a* [12], [13], [16], [17]. Although often speculated (e.g. [6]), the relevance for this molecular function in a developmental context has remained unclear. Intriguingly, mice analyzed in our lab that were mutant for *Tbx2*, showed severely hypoplastic lungs, pointing to a possible role of this T-box factor in the regulation of proliferation and/or differentiation during development of this organ.

The architecture of the mammalian lung arises from a complex developmental program in which the tight orchestration of proliferation and differentiation processes assures the formation of an appropriately sized organ with a correct distribution of differentiated cell types for air-conduction and gas-exchange. In the mouse, the conducting airways develop from two primary buds that emerge from the ventral wall of the foregut endoderm at embryonic day (E) 9.5 by iterative rounds of stereotyped outgrowth and branching until E16.5. The gas-exchange units, the alveoli, only arise subsequently to this pseudoglandular stage from the terminal buds until late in postnatal life. Normal morphogenesis and patterning of the bronchial tree critically depends on the underlying mesenchyme that is derived from the splanchnic mesoderm. This mesenchyme is a source of signals that mediate proliferation of epithelial precursors, and direct their correct spatial differentiation. It also gives rise to a number of different cell types, including parabronchial and vascular smooth muscle cells, lipocytes, fibrocytes and endothelial cells. In turn, epithelial signals from the endoderm but also from the mesothelium maintain proliferation of mesenchymal precursors, closing a reciprocal signaling loop that directs outgrowth of the distal epithelial buds (for a recent review see [18]).

Previous work reported the expression of *Tbx2* and *Tbx3* in the mesenchymal compartment of the developing lung, but a functional significance has not been assigned to this expression [19], [20]. Here, we show by loss- and gain-of-function experiments in the mouse that *Tbx2* is required and sufficient to maintain proliferation and inhibit differentiation in the mesenchymal compartment of the developing lung. Expression, organ culture and biochemical assays identify the cell cycle inhibitors encoded by the *Cdkn1a* and *Cdkn1b* genes as direct targets of Tbx2 repressive activity in this developmental program *in vivo*. Lung growth was substantially rescued by genetically limiting *Cdkn1a* and *Cdkn1b* expression in *Tbx2*-deficient mice, indicating that suppression of these genes is a critical function of Tbx2 in the control of organ growth during development.

## Results

### *Tbx2*-deficient mice exhibit hypoplastic lungs

**Figure 1:**
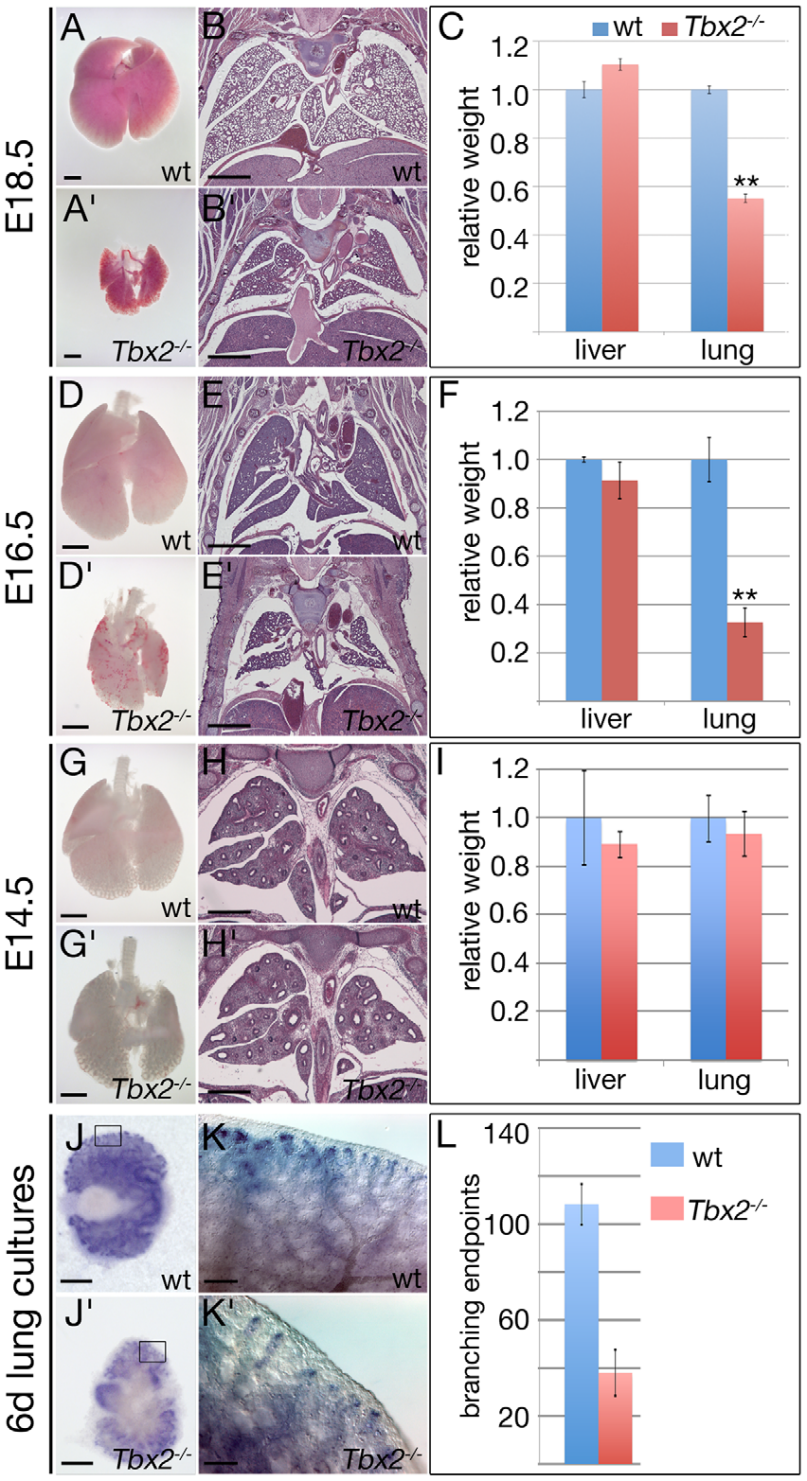
*Tbx2*-deficient lungs become hypoplastic at the late pseudoglandular stage. (A,A′,D,D′,G,G′) Ventral views of whole isolated lungs, (B,B′,E,E′,H,H′) histological analysis by haematoxylin and eosin staining of frontal sections of wildtype and *Tbx2^−/−^* embryos, and (C,F,I) statistical analysis of relative lung per body weight; liver was analyzed as a control organ. Reduction of the lung weight to about half of the wildtype value (100%) at E18.5, and a third at E16.5 was statistically highly significant (**) whereas liver weights and lung weights at E14.5 were without significant change in *Tbx2*-deficient embryos. Stages and genotypes are as indicated. (J–L) Analysis of branching morphogenesis of E12.0 lung rudiments cultured for 6 days by *in situ* hybridization of the epithelial tip marker *Id2* (J–K′), and subsequent statistical analysis reveal a highly significant reduction of branching end-points in *Tbx2*-deficient cultures (L). Scale bars represent 1 mm in A,A′,B,B′,D,D′,E,E′, 500 µm in G,G′,H,H′,J,J′ and 100 µm in K,K′. For statistics see Table S1A.

Mice homozygous for a null allele of *Tbx2* (*Tbx2^cre^*) that is maintained on an NMRI outbred background survive embryogenesis but die shortly after birth due to a cleft palate [9], [21]. Morphological and histological examination of mutant embryos at E18.5 revealed hypoplastic lungs that frequently manifested with alveolar haemorrhages. Air was present in the bronchial network but the lung was poorly inflated. Lobulation was normal but all four right lung lobes and the left lung lobe were reduced in size; the tissue appeared thickened. The weight of the mutant lung was reduced to approx. 50% of that of the littermate control whereas the liver and the spleen were unaffected excluding a general growth retardation problem (Figure 1A–1C). At E16.5, the mutant lung was visibly smaller and haemorrhagic. Its weight was reduced to 33% of the wildtype level (Figure 1D–1F). No obvious difference in morphology, histology and weight of the lung between wildtype and *Tbx2*-deficient embryos was observed at E14.5 (Figure 1G–1I).

To evaluate whether the decreased size of *Tbx2*-deficient lungs after E14.5 relates to a reduction in branching morphogenesis, we explanted E11.5 lung rudiments and analyzed their (2-dimensional) outgrowth after 6 days of culture. Whole-mount *in situ* hybridization analysis for expression of the epithelial tip marker gene *Id2* showed an almost 3-fold reduction of branching endpoints in the *Tbx2*-mutant lung explants suggesting that epithelial branching morphogenesis is indeed severely hampered by loss of *Tbx2* (Figure 1J–1L). However, reduction of branching morphogenesis was restricted to the late phase of lung outgrowth as revealed by non-significant changes of the number of branching endpoints in *Tbx2*-deficient cultures at 2 and 4 days (Figure S1). We conclude that *Tbx2* is required to maintain normal branching morphogenesis and growth of the developing lung after E14.5.

### Decrease in proliferation of mesenchymal progenitor cells

**Figure 2:**
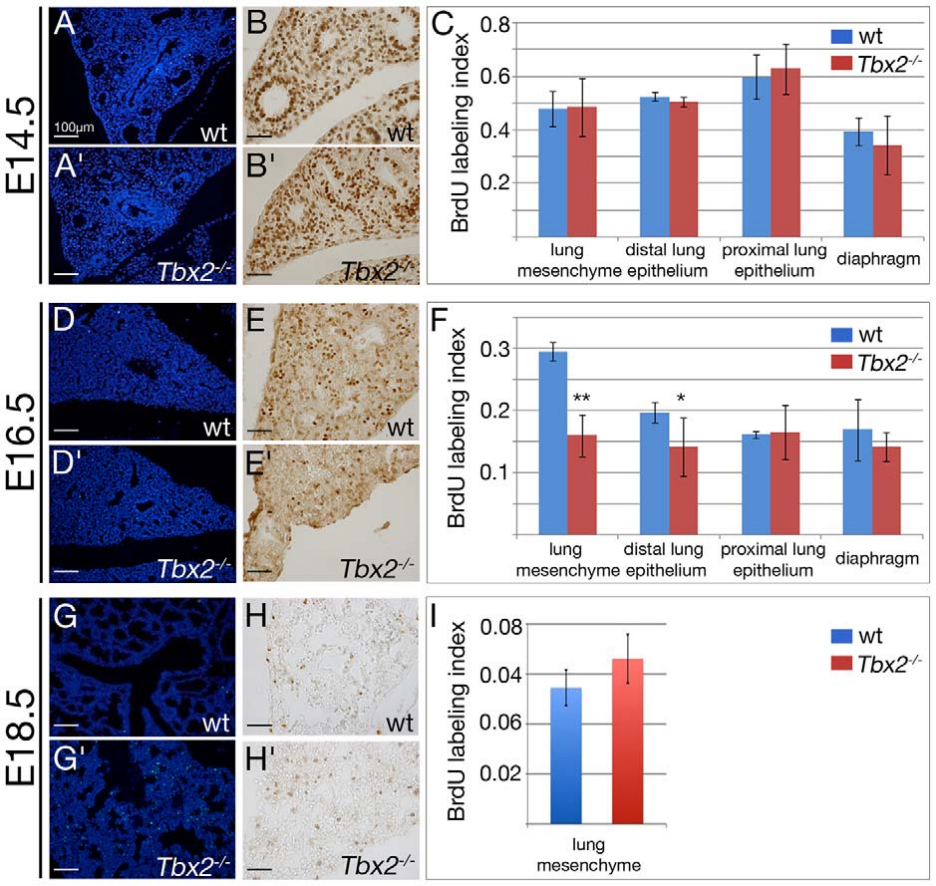
Decreased proliferation and increased apoptosis contribute to hypoplasia of *Tbx2*-deficient lungs. (A,A′,D,D′,G,G′) Analysis of apoptosis by terminal deoxynucleotidyl transferase-mediated nick-end labeling (TUNEL), and (B,B′,E,E′,H,H′) of proliferation by anti-BrdU immunohistochemistry. (C,F,I) Statistical analysis of the BrdU labeling index for the lung mesenchyme, the proximal and distal epithelium, and the diaphragm at different developmental stages. Genotypes and stages are as indicated. Scale bars represent 100 µm in A,A′,D,D′,G,G′, and 50 µm in B,B′,E,E′,H,H′. For statistics see Table S1B.

Lung growth during the pseudoglandular stage is driven by branching morphogenesis of the distal lung buds. This, in turn, relies on rapid proliferation of the precursor cells in the bud epithelium and its underlying mesenchyme. Reduced size of *Tbx2*-deficient lungs could therefore relate to increased apoptosis and/or to decreased proliferation of distal epithelial and mesenchymal tissue compartments as shown for other models of lung hypoplasia [22]. Terminal deoxynucleotidyl transferase-mediated nick-end labeling (TUNEL) staining revealed that apoptosis was absent both in wildtype and mutant lungs at E14.5 and E16.5 but was increased in *Tbx2*-deficient lungs at E18.5 indicating a late contribution to the hypoplasia of this organ (Figure 2A and 2A′, 2D and 2D′, 2G and 2G′).

Analysis of 5-bromo-2′-deoxyuridine (BrdU) incorporation showed that the epithelial and mesenchymal tissue compartments of the lung were highly proliferative irrespective of the genotype at E14.5 (Figure 2B–2C). However, at E16.5 the BrdU labeling index showed a highly significant reduction from 29.6+/−1.5% in the wildtype to 16.0+/−3.4% in the mutant mesenchyme, and a significant reduction from 19.7+/−1.6% in the wildtype to 14.2+/−4.7% in the mutant distal lung epithelium (marked by expression of SRY-box containing protein (Sox)9 [23], [24]) while the proximal lung epithelium (marked by expression of Sox2 [25], [26]) or a control tissue (the diaphragm) were unaffected (Figure 2E–2F, Figure S2). At E18.5, proliferation as indicated by the BrdU labeling index was dramatically decreased in the whole lung to levels similar in wildtype and mutant embryos (Figure 2H–2I). Thus, *Tbx2* is required to maintain normal proliferation of the mesenchyme and distal epithelium of the lung in a narrow temporal window.

### Premature differentiation of the *Tbx2*-deficient lung mesenchyme

**Figure 3:**
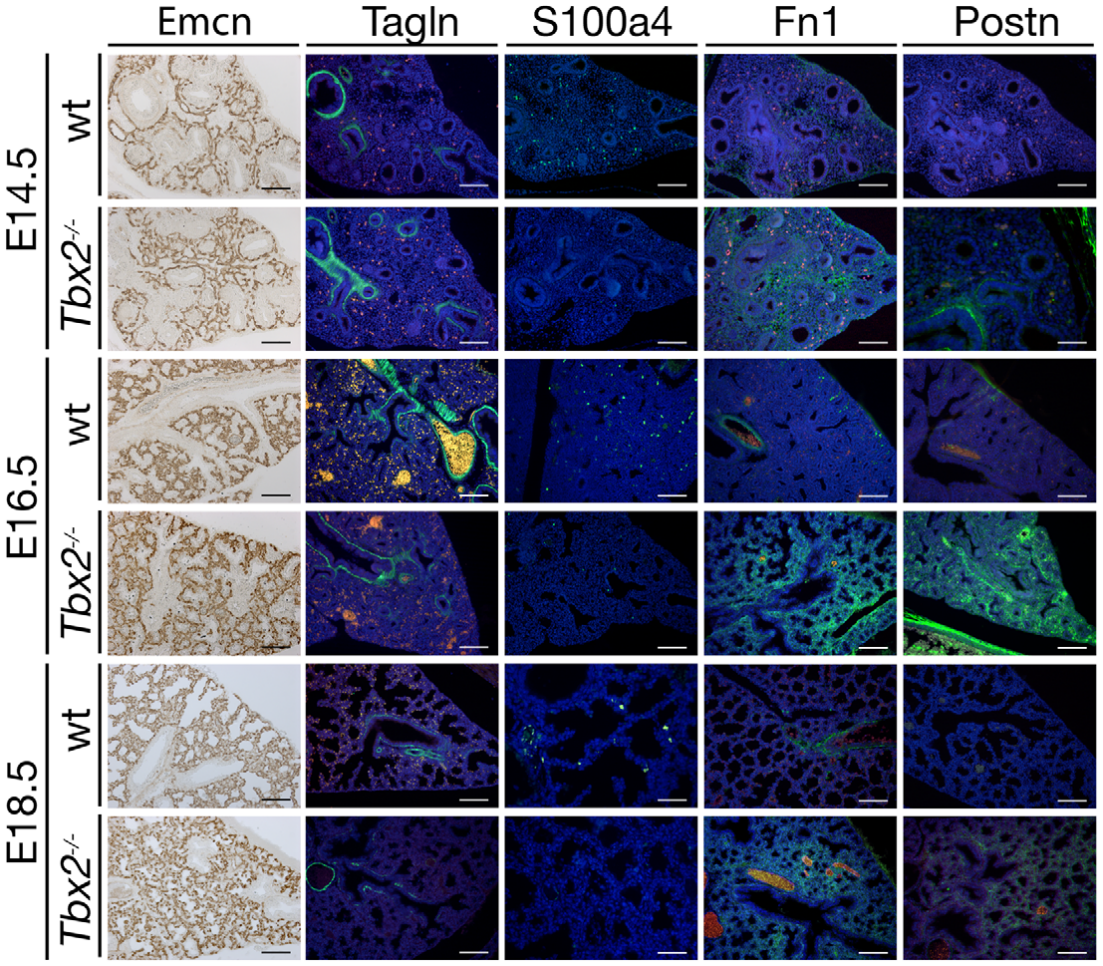
Precocious differentiation in the *Tbx2*-deficient lung mesenchyme. Immunohistochemistry for the endothelial marker Emcn and immunofluorescence analysis for smooth muscle cells (Tagln), immature fibroblasts (S100a4) and fibrocyte-secreted extracellular matrix proteins (Fn1, Postn) on frontal sections of wildtype (wt) and *Tbx2*-deficient (*Tbx2^−/−^*) lungs at E14.5, E16.5 and E18.5 as indicated. S100a4 is lost in *Tbx2*-deficient lungs whereas deposition of Fn1 and Postn is greatly increased. Scale bars represent 100 µm.

As proliferation and differentiation are often inversely correlated, we next investigated the occurrence of changes in the differentiation patterns of both mesenchyme and epithelium in *Tbx2*-deficient lungs at E14.5, E16.5 and E18.5 to cover the period before, around and after the histological and cellular defects were apparent in the mutant (Figure 3). At all analyzed stages a normal distribution of networks of endomucin (Emcn)-positive endothelial cells [27] was present throughout the mutant lung. In the mutant mesenchyme, transgelin (Tagln)-positive smooth muscle cells were restricted to the proximal airways as in the wildtype. It has recently been shown that expression of S100 calcium binding protein A4 (S100a4) marks fibroblasts that are highly proliferative and express low levels of extracellular matrix proteins indicating the precursor character of these cells [28]–[30]. We found that in E14.5 wildtype lungs all mesenchymal cells that were positive for S100a4 also incorporated BrdU confirming the proliferative character of this cell type (Figure S3). In the *Tbx2*-deficient lung, expression of S100a4 was completely abolished. Fibronectin (Fn) and periostin (Postn), extracellular matrix proteins that are secreted by mature fibrocytes at low levels in proximal airways in the wildtype [30]–[32], were expressed throughout the mesenchyme starting from E14.5 (Fn) and E16.5 (Postn) in the mutant lung (Figure 3). This suggests that *Tbx2* is required to maintain the precursor state of a subpopulation of future fibrocytes in the lung mesenchyme.

We next investigated whether these mesenchymal changes are accompanied by alterations in proximal-distal patterning of the respiratory tree and cell differentiation in the epithelium during development in *Tbx2*-deficient lungs (Figure S4). In the wildtype lung, Sox2 was expressed in the trachea and proximal airways and was excluded from distal endoderm at all analyzed stages. Sox9 was expressed in the distal tip endoderm and excluded proximally at E14.5 and E16.5. At E18.5, Sox9 was downregulated distally and reactivated in the mesenchyme of the proximal airways possibly indicating onset of cartilage formation in this region. Expression of keratin 14 (Krt14, also known as cytokeratin 14) in basal cells in the trachea, of tubulin, beta 4A class IVA (Tubb4a) in ciliated cells, and of secretoglobin, family 1A, member 1 (Scgb1a1, also known as CC10 and uteroglobin) in secretory or Clara cells was activated at E16.5 and maintained at E18.5. Krt14 was also found in myofibroblasts surrounding the proximal airways at E16.5. Surfactant associated protein C (Sftpc1, also known as SP-C) was expressed in alveolar epithelial cells type II (AEC2) from E16.5. Expression of podoplanin (Pdpn) and aquaporin 5 (Aqp5) was activated in AEC1 at E18.5. All of these markers (described in [18]) were appropriately activated and maintained in *Tbx2*-deficient lungs with the exception of Pdpn and Aqp5 that showed reduced expression levels at E18.5. We conclude that mesenchymal loss of *Tbx2* does not affect proximal-distal patterning of the lung epithelium. Reduced or delayed differentiation of AEC1 from AEC2 may relate to the loss of appropriate signaling from the prematurely differentiated mesenchyme.

### Coexpression of T-box genes in the developing lung mesenchyme

**Figure 4:**
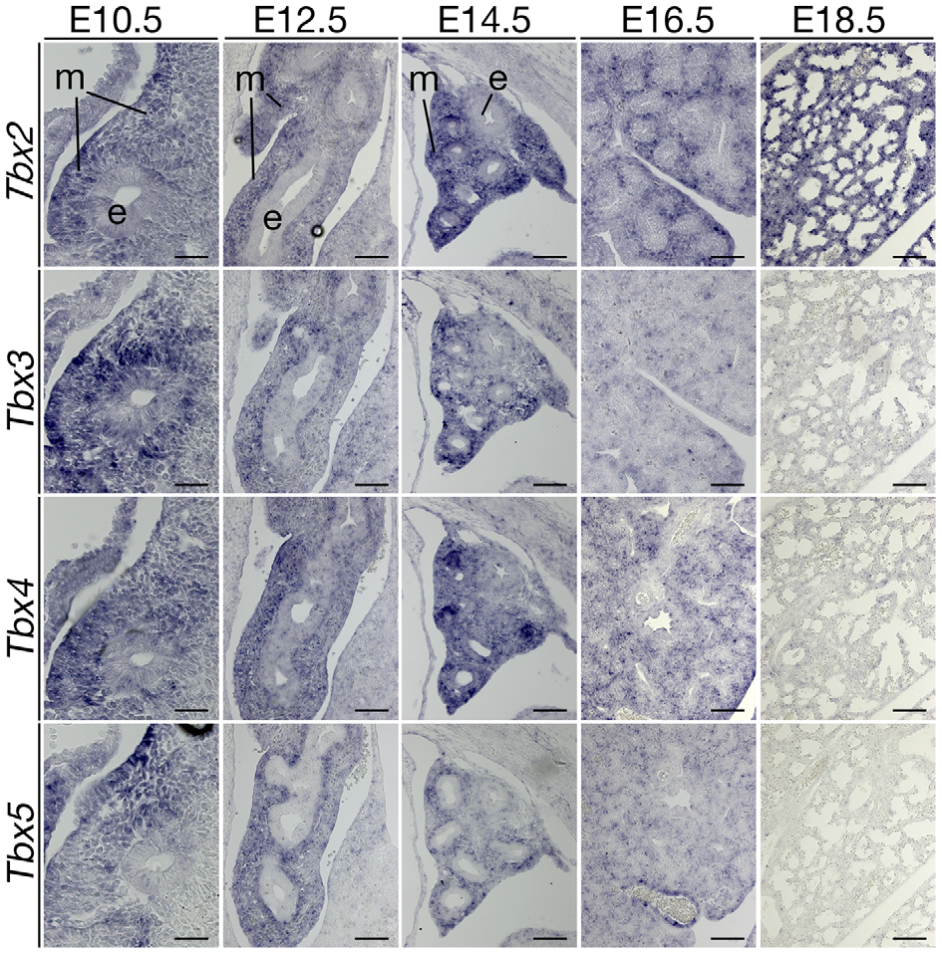
Expression analysis of T-box genes suggests a non-redundant role of *Tbx2* in late lung development. Analysis of *Tbx* gene expression during lung development by RNA *in situ* hybridization on serial sagittal sections of wildtype embryos. The closely related genes *Tbx2* and *Tbx3* are co-expressed until E14.5. Thereafter, only *Tbx2* expression is maintained in the lung mesenchyme. Developmental stages and probes are as indicated in the figure. e, epithelium; m, mesenchyme. Scale bars represent 50 µm at E10.5 and E12.5, 100 µm at E14.5 through E18.5.

Phenotypic changes of *Tbx2*-deficient lungs were confined to the late phase of branching morphogenesis suggesting a narrow temporal window of expression and/or activity of this gene. Alternatively, *Tbx2* may act redundantly with other T-box genes during early lung development. In fact, previous work reported expression of *Tbx2* as well as of *Tbx3*, *Tbx4* and *Tbx5* in the pulmonary mesenchyme [20], [33]. To assess the comparative temporal expression patterns of these genes during lung development, we performed *in situ* hybridization analysis on sagittal sections of the lung (Figure 4). We observed coexpression of *Tbx2* and *Tbx3* in the mesenchymal compartment from E10.5 to E14.5. Expression of *Tbx3* declined sharply after this stage, whereas *Tbx2* was maintained at high levels at subsequent embryonic stages. Coexpression of *Tbx4* and *Tbx5* was found between E10.5 to E16.5 in the lung mesenchyme. Hence, late onset of phenotypic changes in *Tbx2*-deficient lungs may relate to functional redundancy with the closely related *Tbx3* gene during the initial phase of branching morphogenesis. This notion is supported by the finding that mice homozygous for a null allele of *Tbx3* exhibit lungs morphologically and histologically indistinguishable from the wildtype at E14.5, shortly before these mice die ([8] and data not shown). Since mice with more than two mutant alleles of *Tbx2* and *Tbx3* die around E9.5 due to cardiac defects [10], analysis of the functional redundancy of the two genes in early lung development was not possible with the mouse lines available to us.

### De-repression of cell cycle inhibitors in *Tbx2*-deficient lung mesenchyme

An antisense oligonucleotide approach with cultured lung rudiments and more recently conditional gene targeting demonstrated a requirement for mesenchymal *Tbx4* and *Tbx5* in the regulation of pulmonary branching morphogenesis [19], [33]. *Tbx4* and *Tbx5* genetically interact with *Fgf10* during lung growth and branching, and may direct transcriptional activation of *Fgf10* that encodes a potent growth factor in the lung but also in other developmental contexts [33], [34]. Given the molecular nature of Tbx2 and Tbx3 as transcriptional repressors, Tbx2 and Tbx3 may compete with Tbx4 and Tbx5 for binding to conserved DNA-binding sites in the promoter of *Fgf10*, similar to the antagonistic control of *Nppa* expression in the heart by Tbx5 and Tbx2/Tbx3 [35].

**Figure 5:**
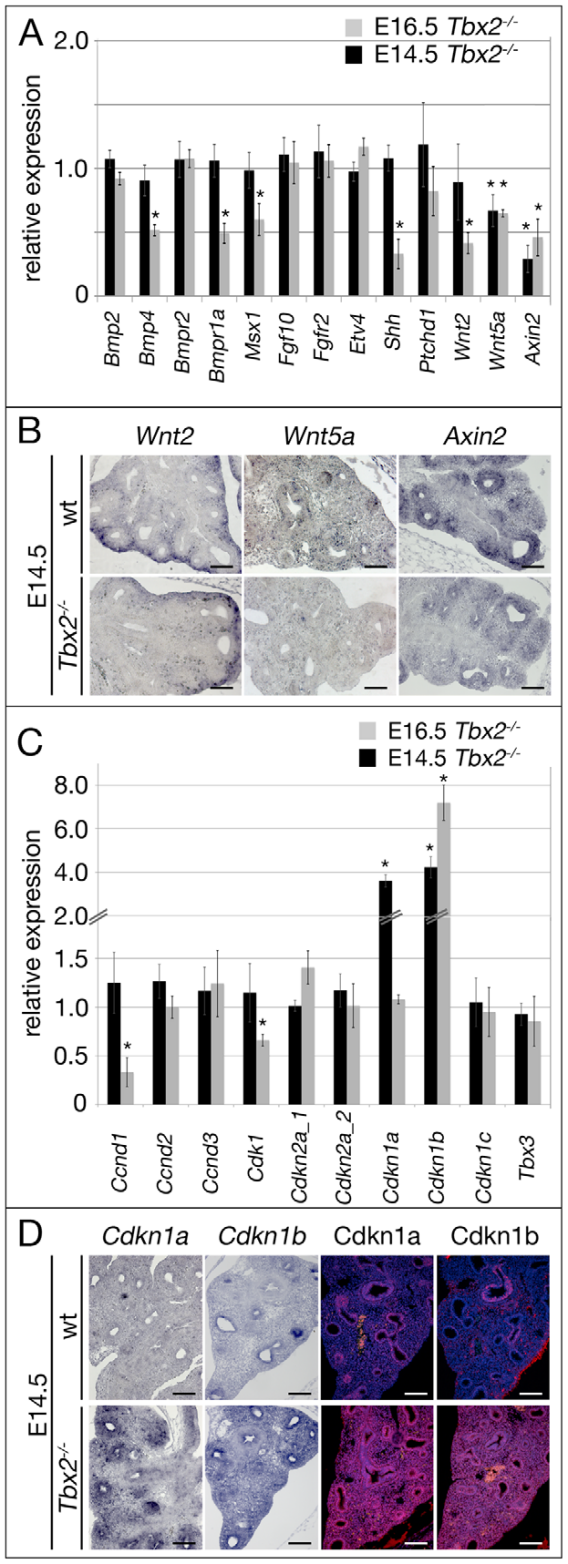
Derepression of genes encoding cell cycle inhibitors precedes proliferation and differentiation defects in the *Tbx2*-deficient lung mesenchyme. (A,C) Semi-quantitative RT-PCR analysis for expression of genes encoding targets and components of Bmp, Fgf, Shh and Wnt pathways that collectively confer outgrowth and branching morphogenesis of the respiratory tree (A), of cell cycle regulators that influence proliferation of the pulmonary tissue and of *Tbx3* to check for compensatory regulation (C) on mRNA harvested from wildtype and *Tbx2*-deficient lungs at E14.5 and E16.5. (B,D) Analysis of expression of *Wnt2*, *Wnt5a*, *Axin2* by *in situ* hybridization (B), and of *Cdkn1a*/Cdkn1*a* and *Cdkn1*/Cdkn1b by *in situ* hybridization and immunofluorescence (D) on frontal sections of lungs at E14.5 in wildtype (wt) and *Tbx2^−/−^* lungs. Scale bars in B and D represent 100 µm. For statistics see Table S1C.

To test this hypothesis and determine the molecular changes underlying the lung phenotype, we analyzed components as well as targets of bone morphogenetic protein (Bmp)-, fibroblast growth factor (Fgf), sonic hedgehog (Shh) and wingless-related MMTV integration site (Wnt) pathways that collectively confer outgrowth and branching morphogenesis of the respiratory tree [18]. To accurately identify expression changes we used quantitative RT-PCR of whole lung extracts at different developmental stages. We started our analysis with lungs at E16.5, when morphological, histological and proliferation defects were fully apparent (Figure 5A, grey bars). At this stage, we observed a significant downregulation of components of the Bmp pathway such as *Bmp4* and *Bmp receptor (Bmpr)2* as well as the Bmp target gene *homeobox*, *msh-like (Msx)1* [36]. *Bmp2* and *Bmpr1a* expression, however, was not significantly altered. Expression of *Shh* was markedly reduced but not accompanied by decreased intracellular signaling as revealed by almost normal expression of the target gene *patched (Ptch)1* [37]. Wnt ligands *Wnt2* and *Wnt5a* were strongly reduced in their expression as was the target of the canonical (Ctnnb1-dependent) sub-branch of Wnt signaling, *Axin2* [38]. Unexpectedly, no changes in Fgf pathway components were found. *Fgf10* expression was at wildtype level as was the receptor *Fgfr2* and the known Fgf target *ets variant gene 4* (*Etv4*, also known as *Pea3*) [39]. At E14.5, i.e. prior to the observed phenotypic changes, components and targets of Shh-, Fgf- and Bmp-activity were unchanged in their expression. The canonical Wnt target gene *Axin2* and the non-canonical ligand *Wnt5a* were strongly and *Wnt2* expression was slightly reduced in mutant lungs (Figure 5A, black bars). *In situ* hybridization analysis showed that downregulation of *Wnt2*, *Wnt5a* and *Axin2* was confined to the mesenchymal compartment of E14.5 *Tbx2^−/−^* lungs (Figure 5B). These data suggest, that Tbx2 does not counteract the transcriptional activation of *Fgf10* transcription and Fgf signaling by Tbx4/Tbx5 but targets canonical Wnt signaling in the lung mesenchyme, what, in turn, may secondarily affect Bmp signaling.

Next, we analyzed expression of cell cycle regulators potentially involved in proliferation control of lung mesenchyme (Figure 5C, grey bars). Among the tested cell cycle activators *cyclin-dependent kinase (Cdk)1* and *cyclin D (Ccnd)1* showed significant reduction whereas *Ccnd2* and *Ccnd3* expression was unchanged at E16.5. As *Ccnd1* has been described as target of canonical Wnt signaling [40], its reduced expression may relate to the observed downregulation of this pathway. The cell cycle inhibitors *Cdkn1a*, *Cdkn1c*, *Cdkn2a* and *Cdkn2d* were unchanged whereas *Cdkn1b* was upregulated more than 7 times in the mutant at this stage. At E14.5, all cell cycle regulators were unaffected except *Cdkn1b* and *Cdkn1a* that were upregulated 4 and 3.5 times, respectively, in the *Tbx2*-deficient lung (Figure 5C, black bars). Expression of *Tbx3* was unaltered at both analyzed stages excluding a compensatory upregulation of this gene in the *Tbx2*-mutant background (Figure 5C). *In situ* hybridization and immunofluorescence analyses confirmed strong upregulation of *Cdkn1a/Cdkn1b* mRNA and Cdkn1a/Cdkn1b protein both in the mesenchymal and in the distal epithelial compartment of E14.5 *Tbx2^−/−^* lungs (Figure 5D). These results argue that reduced proliferation in the mesenchyme and distal epithelium (that are probably secondary to altered mesenchymal signals) of E16.5 *Tbx2*-deficient lungs may be caused by de-repression of cell cycle inhibitors *Cdkn1a* and *Cdkn1b*. Decreased (canonical) Wnt signaling in *Tbx2*-deficient lungs may reflect an independent branch of Tbx2 activity, or may merely present a secondary consequence of de-repression of cell cycle inhibitors.

### Repression of *Cdkn1a* and *Cdkn1b* by Tbx2 is direct and contributes to lung growth

**Figure 6:**
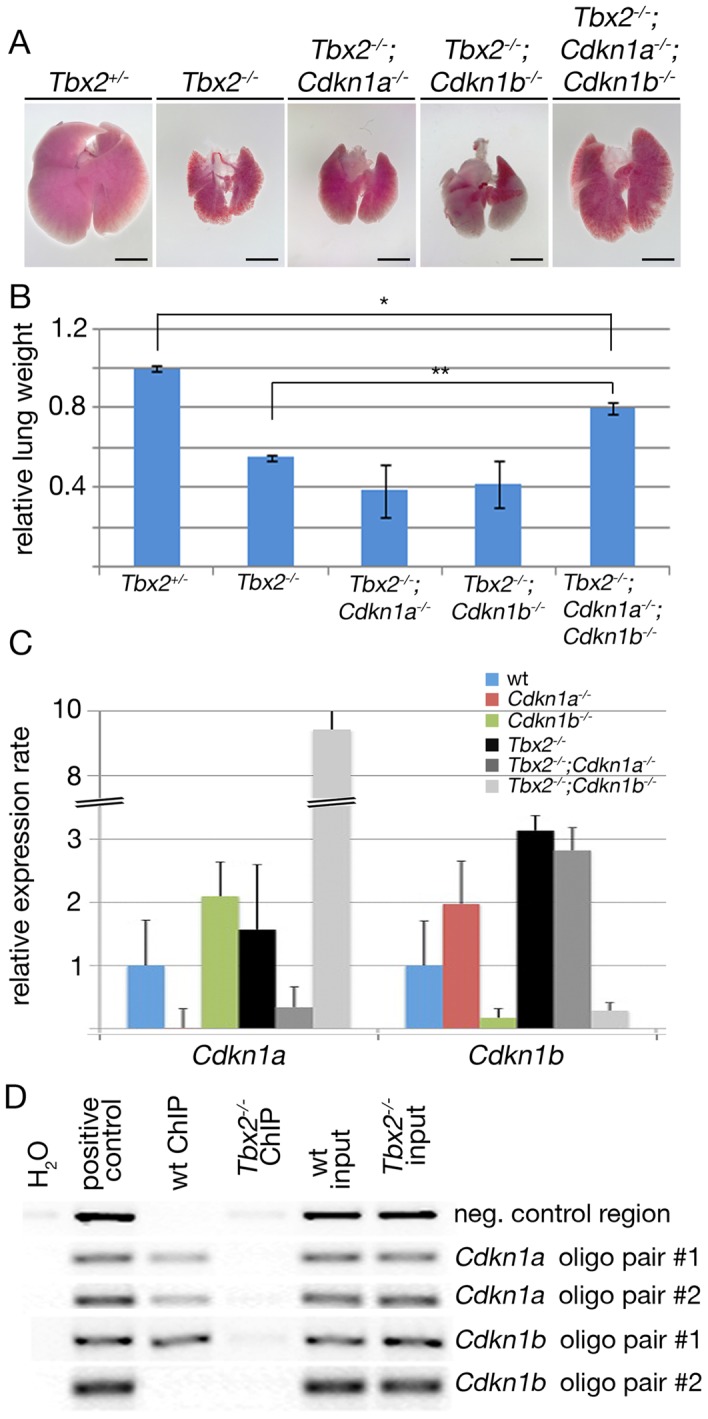
*Cdkn1a* and *Cdkn1b* are direct targets of Tbx2 in the lung mesenchyme. (A,B) Morphological (A) and quantitative analysis (B) of E18.5 lungs of compound mutants of *Tbx2*, *Cdkn1a* and *Cdkn1b*. Shown are relative lung weights (lung weight to body weight with the control *Tbx2^+/−^* set to 1). * indicates significant, ** highly significant changes. Scale bars in A represent 2.5 mm. (C) Semi-quantitative RT-PCR analysis for expression of *Cdkn1a* and *Cdkn1b* in lungs compound for *Tbx2-*, *Cdkn1a-* and *Cdkn1b*-null alleles. Expression levels are represented as fold changes with respect to the wildtype that was set to 1. (D) ChIP analysis of Tbx2 binding to putative T-box binding elements (TBEs) in the *Cdkn1a* and *Cdkn1b* locus, respectively. H_2_O refers to a control containing no DNA, positive control to genomic DNA, wt ChIP to chromatin of wildtype lungs, and *Tbx2^−/−^* ChIP to chromatin of *Tbx2* ^−/−^ lungs immunoprecipitated with an anti-TBX2 antibody; wt input and *Tbx2^−/−^* input refers to chromatin before the immunoprecipitation step. Five genomic elements were tested: a negative control region without TBE downstream of the *Cdkn1b* locus, a region from the 5′ UTR of the *Cdkn1a* enclosing the sequence AGGTGTGA amplified with the *Cdkn1a* primer pair #1 and an alternative primer pair #2, an element 3 kbp upstream of the start codon in the *Cdkn1b* locus harboring the site AGGTGTGTG amplified with the primer pair #1, and an intronic element in the *Cdkn1b* locus harboring an inverse TBE site CACACCT amplified with the primer pair #2. For statistics see Table S1D.

To unravel the contribution of increased expression of *Cdkn1a* and *Cdkn1b* to the growth deficit of *Tbx2-*deficient lungs, we ablated the two genes in the mutant background. Compound *Tbx2*;*Cdkn1a* and *Tbx2*;*Cdkn1b* mutants, respectively, exhibited lungs that were morphologically indistinguishable from the *Tbx2*-single mutant organ. In contrast, triple *Tbx2*;*Cdkn1a*;*Cdkn1b* mutants exhibited visibly larger lungs at E18.5 (Figure 6A). To quantify the observed changes, we determined the relative lung weight (lung weight to body weight ratios, normalized to that of *Tbx2^+/−^* control embryos) of the different compound mutants. Statistical analysis did not detect significant weight changes between *Tbx2^−/−^*;*Cdkn1a^−/−^* and *Tbx2^−/−^*;*Cdkn1b^−/−^* lungs, whereas the increase in weight in *Tbx2^−/−^*;*Cdkn1a^−/−^*;*Cdkn1b^−/−^* lungs was highly significant (Figure 6B). Although *Tbx2^−/−^*;*Cdkn1a^−/−^*;*Cdkn1b^−/−^* lungs reached 80% of the control weight, the difference remained significant indicating an incomplete rescue. This suggests that the combined de-repression of *Cdkn1a* and *Cdkn1b* accounts predominantly but not completely for hypoplasia of *Tbx2*-deficient lungs.

Since the individual deletion of *Cdkn1a* and *Cdkn1b* in the *Tbx2*-mutant background did not lead to even a partial rescue of growth, we tested for the presence of a compensatory mechanism by analyzing expression of *Cdkn1a* and *Cdkn1b*, respectively, by quantitative RT-PCR analysis on mRNA of E16.5 (compound) mutant lungs (Figure 6C). *Cdkn1a* expression was increased 2-fold in *Cdkn1b^−/−^* lungs and 9-fold in *Tbx2^−/^*;*^−^Cdkn1b^−/−^* lungs whereas *Cdkn1b* was increased 2-fold in *Cdkn1a^−/−^* lungs and 3-fold in *Tbx2^−/−^*;*Cdkn1a^−/−^* lungs at this stage. Thus, either gene shows a compensatory upregulation upon loss of the other gene.

Binding sites for TBX2 within the *Cdkn1a* promoter have recently been described in cell culture experiments [12] whereas *Cdkn1b* has not been recognized as a direct target of Tbx2 repressive activity before. *In silico* analysis of the mouse *Cdkn1a* and *Cdkn1b* genes identified a consensus DNA-binding site for T-box proteins (T-box binding element (TBE): AGGTGTGA) [41] in the *Cdkn1a* promoter and two putative TBEs in the *Cdkn1b* locus. The first element (AGGTGTGTG) was detected 3 kbp upstream of the start codon, the second element with the reverse complementary sequence CACACCT was localized within an intron of that gene (Figure S5). ChIP experiments with E15.5 lung tissue revealed *in vivo* binding of Tbx2 to the known TBE in the *Cdkn1a* locus and to the 5′ located but not the intronic TBE in the *Cdkn1b* gene (Figure 6D) compatible with the notion that *Cdkn1a* and *Cdkn1b* represent direct targets of Tbx2 repressive activity in the lung mesenchyme.

It has previously been shown that Cdkn1a expression is elevated on inactivation of endogenous *Tbx2* in the murine B16 melanoma and the human MCF-7 breast cancer cell line [12]. Using the previously published conditions [12], we downregulated *Tbx2* in both cell lines using a *Tbx2*-specific siRNA approach. Immunofluorescence analysis showed that in the non-silencing control nuclear Tbx2 protein was present in all cells whereas Cdkn1b was not detected. In contrast, in cells treated with the *Tbx2*-specific siRNA Tbx2 expression was extinguished in almost all cells examined whereas Cdkn1b expression was strongly upregulated in the cytoplasm (in MCF-7 cells) and in the nucleus (in B16 melanoma cells) (Figure S6). This further supports that *Cdkn1b* similar to *Cdkn1a* is a true target of Tbx2.

### Maintenance of Tbx2 expression prevents terminal differentiation of lung fibrocytes

**Figure 7:**
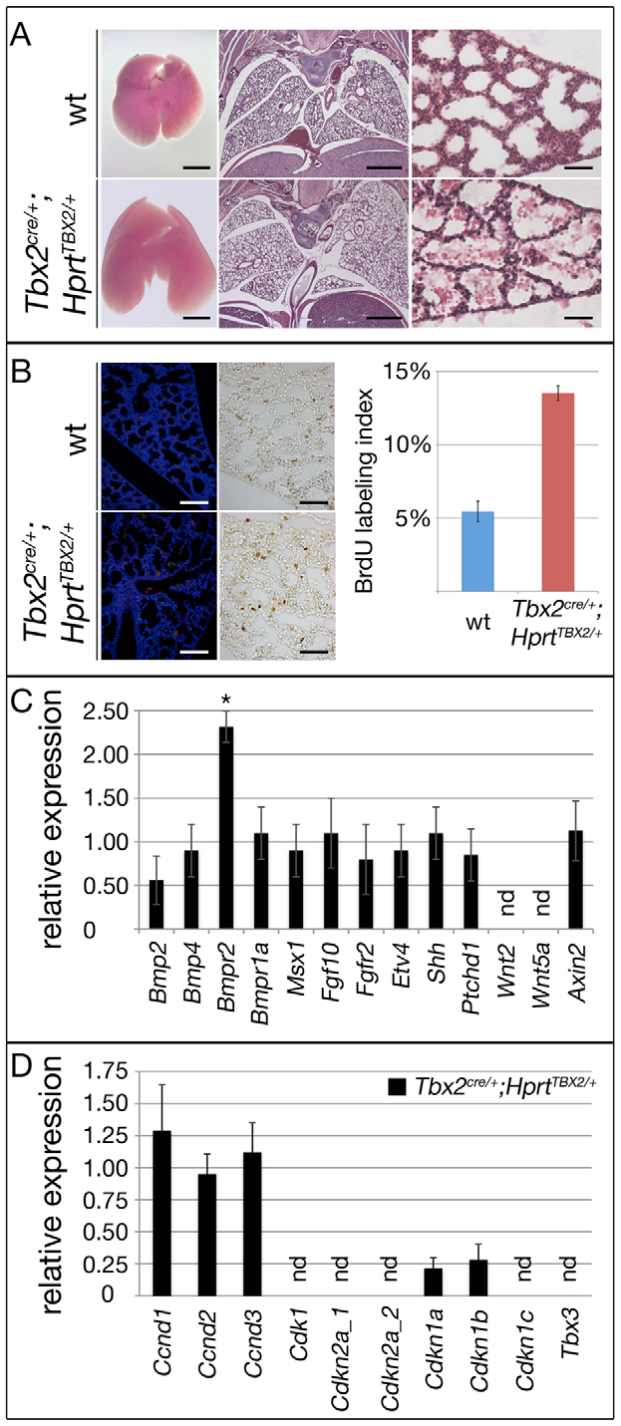
Prolonged expression of TBX2 maintains proliferation of mesenchymal progenitor cells in the embryonic lung. Analysis of lungs of wildtype and *Tbx2^cre/+^*;*Hprt^TBX2/+^* mice at E18.5. (A) Morphology of whole lungs and histological analysis by haematoxylin and eosin (HE) staining of frontal lung sections. (B) Analysis of apoptosis by TUNEL staining and of proliferation by the BrdU incorporation assay on frontal sections of the lung. TBX2-overexpressing mice show highly increased BrdU incorporation. (C and D) Semi-quantitative RT-PCR analysis for gene expression in *Tbx2^cre/+^*;*Hprt^TBX2/+^* lungs at E18.5. Expression levels are represented as fold changes with respect to the wildtype that was set to 1. Overexpression of TBX2 does not affect signaling (C) but leads to downregulation of *Cdkn1a* and *Cdkn1b* in the lung (D). Scale bars in B represent 100 µm for TUNEL and 50 µm for anti-BrdU assays. For statistics see Table S1E.

To further evaluate the mechanistic role of Tbx2 in the lung mesenchyme, we additionally employed an *in vivo* gain-of-function approach. For this, we crossed the *Tbx2^cre^* line and an *Hprt^TBX2^*-allele, that was generated by integration of a bicistronic transgene-cassette containing the human *TBX2* ORF followed by *IRES-GFP* in the ubiquitously expressed X-chromosomal *Hypoxanthine guanine phosphoribosyl transferase* (*Hprt*) locus [10], [42] to maintain *TBX2* expression in its endogenous domains including the lung mesenchyme. Male (*Tbx2^cre/+^*;*Hprt^TBX2/y^*) embryos were not recovered after E12.5 most likely due to cardiac defects. In contrast, female (*Tbx2^cre/+^*;*Hprt^TBX2/+^*) embryos, which exhibit a mosaic expression due to random X-chromosome inactivation, survived embryogenesis and puberty. Lungs of E18.5 *Tbx2^cre/+^*;*Hprt^TBX2/+^* embryos were slightly bigger than those of control littermates and showed a looser tissue organization (Figure 7A). Apoptosis was not detected in either genotype, but *Tbx2^cre/+^*;*Hprt^TBX2/+^* lungs exhibited a strong increase of proliferation in the mesenchyme as shown by the BrdU assay (Figure 7B). Notably, Western blot analysis of lungs of E18.5 *Tbx2^cre/+^*;*Hprt^TBX2/+^* embryos showed that transgenic TBX2 expression did not reach unphysiologically high levels (Figure S7).

Branching morphogenesis is downregulated after E16.5 concomitant with the shut-down of signaling pathways involved in epithelial-mesenchymal tissue interactions at the distal lung buds. Therefore, increased proliferation in *Tbx2^cre/+^*;*Hprt^TBX2/+^* lungs may relate to continued branching by maintained activity of these signaling pathways. Morphological inspection did not detect changes of branching between E12.0 wildtype and *Tbx2^cre/+^*;*Hprt^TBX2/+^* lung rudiments cultured for 6 days (Figure S8). Furthermore, RT-PCR analysis found unchanged expression of targets of Shh (*Ptch1*), Fgf (*Etv4*), Bmp (*Msx1*) and canonical Wnt (*Axin2*) pathways in *Tbx2^cre/+^*;*Hprt^TBX2/+^* lungs at E18.5 showing that Tbx2 is not sufficient to induce these pathways, thus, branching morphogenesis (Figure 7C). However, when testing cell cycle regulators in this assay, we detected a selective downregulation of *Cdkn1a* and *Cdkn1b* in *Tbx2^cre/+^*;*Hprt^TBX2/+^* lungs showing that Tbx2 is not only required but also sufficient to repress expression of *Cdkn1a* and *Cdkn1b* (Figure 7D).

**Figure 8:**
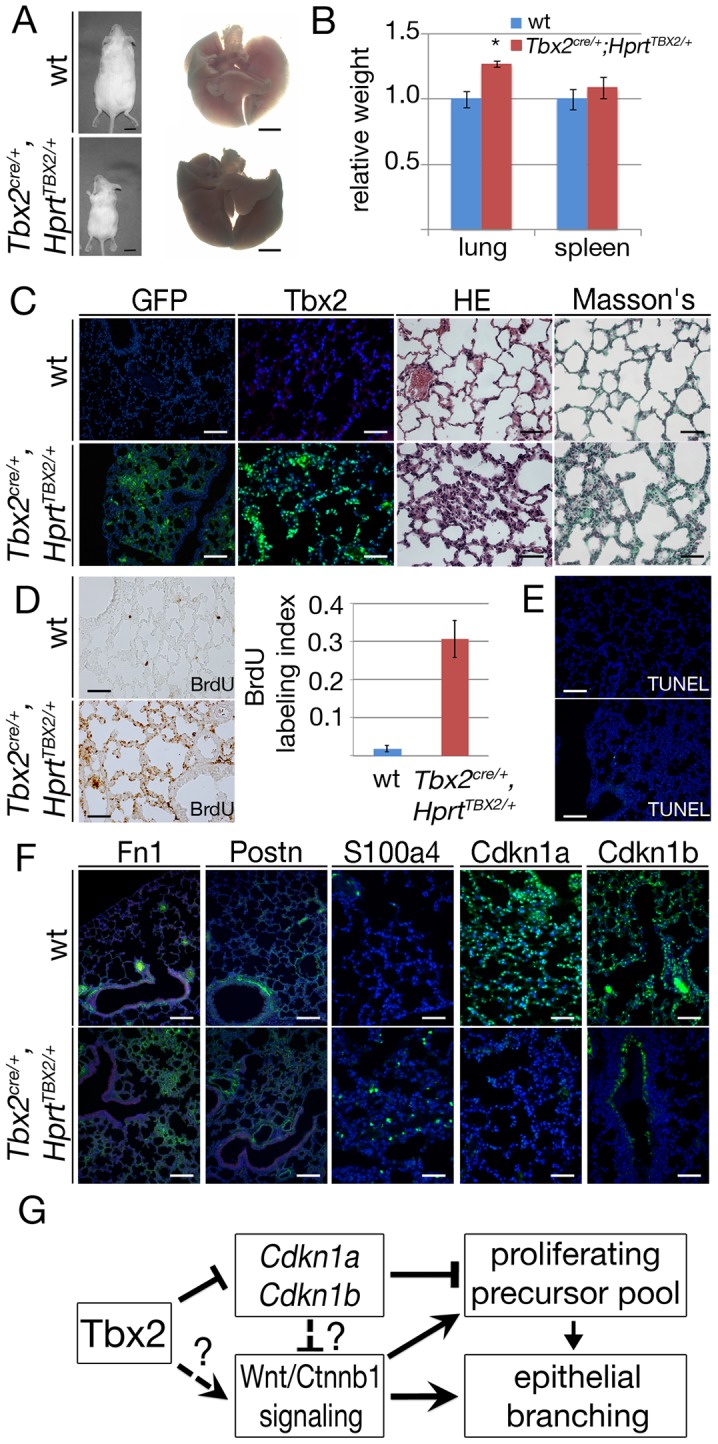
Prolonged expression of TBX2 maintains proliferation of mesenchymal progenitor cells in the adult lung. Analysis of wildtype and *Tbx2^cre/+^*;*Hprt^TBX2/+^* mice at P40. (A) Morphology of P40 mice and lungs. (B) Statistical analysis of relative lung per body weight; spleen was analyzed as a control organ. Increase of the lung weight in *Tbx2^cre/+^*;*Hprt^TBX2/+^* mice was statistically significant (*). (C) Immunofluorescence analysis of GFP and Tbx2/TBX2 expression, histological analysis by haematoxylin and eosin (HE) and Masson's staining on sections of P40 lungs. (D) BrdU incorporation assay of frontal sections of the lung. TBX2-overexpressing mice show highly increased BrdU incorporation. (E) Analysis of apoptosis by TUNEL staining. (F) Immunofluorescence analysis of Fn1, Postn, S100a4, Cdkn1a and Cdkn1b expression on lung sections at P40. Genotypes are as indicated. Scale bars in A represent 1 cm and 2.5 mm, respectively. Scale bars in C represent 100 µm for GFP, otherwise 50 µm, in D 50 µm, in E 100 µm, in F 100 µm for Fn1 and Postn and for all others 50 µm. For statistics see Table S1F. (G) Model of the role of Tbx2 in lung development.

To evaluate long-term consequences of prolonged TBX2 expression in the lung mesenchyme, we analyzed *Tbx2^cre/+^*;*Hprt^TBX2/+^* mice at postnatal day (P) 40, a stage when they were present in the expected numbers. Although *Tbx2^cre/+^*;*Hprt^TBX2/+^* mice were visibly smaller than their littermate controls at this stage, the relative lung mass was increased by a factor of 1.27 (Figure 8A and 8B). Immunofluorescence for GFP and TBX2 expression on lung sections confirmed the widespread expression of the transgene in the mesenchymal compartment of P40 *Tbx2^cre/+^*;*Hprt^TBX2/+^* mice. Histological analysis by haematoxylin and eosin staining uncovered clusters of tissue thickenings, and alveolar air spaces were surrounded by multiple cell layers in these transgenic lungs. Histological staining for keratin and collagen (Masson's trichrome) did not detect changes in the transgenic lung, excluding the possibility that tissue thickening is caused by excessive deposition of extracellular matrix (Figure 8C). Analysis of BrdU incorporation showed that the lung tissue was highly proliferative in the transgenic animals at P40 (*Tbx2^cre/+^*;*Hprt^TBX2/+^*: 31.0%±5.2, control: 2.1%±0.8) (Figure 8D). Apoptosis as detected by TUNEL staining was similarly absent from control and transgenic lungs (Figure 8E).

Analysis of cell differentiation by immunofluorescence of marker proteins showed normal presence of lung epithelial cell types, of endothelial cells, and of mesenchymal smooth muscle cells around the proximal airways of *Tbx2^cre/+^*;*Hprt^TBX2/+^* lungs (Figure S9). Fn1 and Postn deposition in the extracellular matrix was augmented, and S100a4-positive cells were increased in number. Expression of the cell cycle inhibitors Cdkn1a and Cdkn1b was dramatically downregulated in the mesenchymal compartment (Figure 8F). Together these findings indicate that prolonged expression of TBX2 maintains mesenchymal proliferation at a high level. While a part of these mesenchymal cells differentiate into ECM-producing cell-types, a substantial fraction retains a S100a4-positive precursor character.

To determine the contribution of reduced expression of *Cdkn1a* and *Cdkn1b* to the observed histological, immunohistochemical and molecular changes in *Tbx2^cre/+^*;*Hprt^TBX2/+^* mice, we additionally analyzed *Cdkn1a^−/−^*;*Cdkn1b^−/−^* mice at P40 using a similar panel of assays. Double mutant lungs, normalized against the increased body weight, were significantly larger than lungs of their littermates (Figure S10A, S10B). Histological analysis did not find changes in the tissue organization (Figure S10C) but *Cdkn1a^−/−^*;*Cdkn1b^−/−^* lungs exhibited a 5-fold increase of proliferation as shown by the BrdU assay compared to the wildtype. Apoptosis was unaffected (Figure S10D, S10E). Fn1 and Postn deposition in the extracellular matrix was normal and immature fibroblasts (S100a4) were absent as in the wildtype. Immunofluorescence analysis of Cdkn1a and Cdkn1b confirmed that both proteins were completely absent in the *Cdkn1a^−/−^*;*Cdkn1b^−/−^* lung (Figure S10F). Hence, *Cdkn1a^−/−^*;*Cdkn1b^−/−^* mice do not feature the histological and cellular changes seen in *Tbx2^cre/+^*;*Hprt^TBX2/+^* mice, but exhibit increased lung mass due to increased proliferation. We conclude that downregulation of Cdkn1a and Cdkn1b mediates the pro-proliferative effects of Tbx2 overexpression to a large degree but may not account for changes in tissue architecture and cell differentiation.

## Discussion

Branching morphogenesis and growth of the lung requires the coordination of cellular behaviors of its epithelial and mesenchymal tissue compartments. Here, we have identified Tbx2 as a crucial mesenchymal factor that maintains the mesenchymal signaling center for epithelial branching morphogenesis. We suggest that Tbx2 promotes mesenchymal proliferation and inhibits terminal differentiation partly via direct transcriptional repression of cell cycle inhibitor genes. Irrespective of its precise mode, Tbx2 additionally maintains canonical Wnt signaling in the mesenchyme, which, in turn, may account for maintenance of epithelial growth and branching at the distal tips of the lung buds (Figure 8G).

### Tbx2 directly represses cell cycle regulators in the lung mesenchyme

Cdkn1a, Cdkn1b together with Cdkn1c constitute the Cip/Kip family of CKIs that inhibit cell cycle progression by binding to and inhibition of a broad range of cyclin-CDK complexes via a shared N-terminal cyclin-CDK binding domain. Cdkn1 activity correlates with cell cycle exit and differentiation, and is, thus, under tight control of anti-mitogenic signals. In tissue homeostasis, expression and activity of CKIs is regulated by a large number of molecular mechanisms including protein binding and posttranslational modification that affect cyclin/CDK binding as well as stability and degradation of CKIs (for a review see [43]). Gene targeting experiments have unambiguously shown that all three members are important players in tissue homeostasis and cancer (for a review see [2]) whereas Cdkn1c is the only CKI to be uniquely required for embryonic development [44], [45]. However, additional congenital defects have been described in mice lacking more than one member of this gene family pointing to redundant functions in some but not all developmental processes (see e.g. [46], [47].

To exert a precise timing of cell cycle exit and differentiation in development, expression of *Cdkn1* genes must be tightly controlled on the transcriptional level. In fact, *Cdkn1a* and *Cdkn1c* have specific patterns of expression in development that correlate with terminal differentiation of multiple cell lineages including skeletal muscle, cartilage, skin, and nasal epithelium. In contrast, *Cdkn1b* expression appears more widespread (for a review see [2]. Cell culture experiments identified *Cdkn1a* as a transcriptional target of p53 [48], [49] whereas the transcriptional regulation of *Cdkn1c* is mediated by factors that play critical roles during embryogenesis such as Notch/Hes1, MyoD and p73 [50]–[52]. To our knowledge, the *in vivo* relevance of these regulatory modules has remained unclear. Interestingly, previous efforts were largely directed towards the identification of transcriptional activators of *Cdkn1* genes, and the possibility that these genes are subject to negative regulation *in vivo*, i.e. that activation of expression in a certain cell type results from attenuation or abolition of a prior transcriptional repression, was neglected.

Here, we have shown that *Cdkn1a* and *Cdkn1b* are derepressed in the pulmonary mesenchyme in *Tbx2*-deficient mice prior to other molecular changes, that *Cdkn1a* and *Cdkn1b* are repressed upon ectopic expression of TBX2 in mature lung mesenchyme, and that deletion of *Cdkn1a* and *Cdkn1b* largely rescued the growth defects of *Tbx2*-deficient lungs. Furthermore, we identified by ChIP analysis Tbx2 binding to *Cdkn1a* and *Cdkn1b* loci in the developing lung. Together, our genetic and biochemical analyses provide evidence that *Cdkn1a* and *Cdkn1b* are subject to direct repression by Tbx2 and are crucial downstream mediators of this gene in the mesenchymal compartment of the developing lung. In turn, it is the first clear evidence, that Tbx2 directly regulates cell cycle control genes in a developmental context *in vivo*. Intriguingly, ChIP-seq analysis of genomic binding of Tbx3 in cardiomyocytes *in vivo*, identified a large number of loci with binding peaks containing a variant TBE [53]. Tbx3 and Tbx2 are closely related family members that recognize the same DNA binding site. Re-inspection of this data set identified binding peaks of Tbx3 in both the *Cdkn1a* and *Cdkn1b* loci. In fact, the DNA-element used in our ChIP analysis precisely mapped to a major Tbx3 peak in the promoter of the *Cdkn1a* locus which contained additional less conserved TBEs, whereas the DNA element used for our *Cdkn1b* ChIP located closely to a minor peak (Figure S11). This together with enhanced expression of Cdkn1a and Cdkn1b in melanoma and breast cancer cell lines depleted of endogenous Tbx2 [12], [13], [17] (and this study), indicates that Tbx3 and the closely related Tbx2 protein occupy DNA sites in the *Cdkn1a* and *Cdkn1b* loci in other cell types and may regulate these genes in other developmental contexts as well.

It should be noted that changes of *Tbx2* did not only (inversely) affect proliferation in the lung mesenchyme but directly correlated with the precursor state of at least one mesenchymal sub-population, S100a4-positive fibroblasts. Although *Cdkn1*-mediated cell cycle arrest has been associated with cellular differentiation in different developmental contexts [44], [54], [55], we did not observe differentiation defects in lungs double mutant for *Cdkn1a* and *Cdkn1b*. This may indicate that in this developmental context negative control of cell differentiation by Tbx2 is not mediated by repression of *Cdkn1* and *Cdkn1b*. Changes of *Tbx2* expression did not affect differentiation of other mesenchyme-derived cell types including smooth muscle cells. This may indicate that Tbx2 does not control differentiation of these cell types, or it may simply reflect the fact that these cell types differentiate prior to E14.5 when *Tbx3* expression is downregulated and *Tbx2* is uniquely required. In the future, it will be interesting to study the relation between mesenchymal proliferation and differentiation in mice deficient for both *Tbx2* and *Tbx3*, which are likely to act redundantly throughout the pseudoglandular stage until E14.5.

### Tbx2 maintains canonical Wnt signaling in the lung mesenchyme

Combined deletion of *Cdkn1a* and *Cdkn1b* function in *Tbx2*-deficient embryos restored lung growth largely but not completely suggesting that additional factors or pathways may act downstream of Tbx2 to mediate mesenchymal proliferation. Our RT-PCR analysis of signaling pathways relevant for branching morphogenesis did not detect changes of Fgf and Shh signaling but uncovered reduced activity of canonical Wnt and Bmp signaling. Notably, we detected decreased expression of Wnt components and signaling as early as E14.5 in the pulmonary mesenchyme, whereas *Bmp4* expression and Bmp signaling was unchanged at that stage suggesting a secondary mode of change of the latter. As Bmp4 was shown to act as an autocrine signal for distal endoderm proliferation [56], reduced expression may contribute to the reduced proliferation in the distal endoderm at E16.5 in *Tbx2*-deficient lungs.

A number of studies have implicated different *Wnt* genes in lung development. Mice deficient for the non-canonical Wnt ligand gene *Wnt5a*, which is expressed in the distal lung mesenchyme, exhibit increased cell proliferation in both epithelium and mesenchyme with a resulting expansion of the distal lung and increased lung size [57]. Wnt2 is a canonical Wnt ligand robustly expressed in the mesenchyme of the developing lung. *Wnt2*, in cooperation with *Wnt2b*, is essential for specification of the respiratory lineage in the anterior foregut endoderm [58]. Later, Wnt2 acts upstream of *Fgf10* and the critical transcription factor myocardin to regulate early airway smooth muscle cell differentiation in the multipotent lung mesenchyme [59]. Finally, Wnt7b, a canonical ligand expressed in the pulmonary epithelium stimulates embryonic lung growth by increasing proliferation in both tissue compartments of the developing lung without affecting the differentiation patterns [22]. Furthermore, tissue-specific deletion of the unique signaling mediator of the canonical pathway, *Ctnnb*1, in the epithelium led to defects in proximal-distal differentiation of airway epithelium [60] whereas mesenchymal deletion of *Ctnnb1* resulted in hypoplasia due to reduced epithelial and mesenchymal proliferation [61].

Maintained differentiation of airway smooth muscle cells but decreased proliferation in the epithelial and mesenchymal compartments during the late phase of branching morphogenesis is compatible with the idea that loss of Tbx2 affects the canonical Wnt pathway in the mesenchyme triggered by the epithelial Wnt7b signal. The growth-promoting effect of this pathway may at least partly be mediated by activation of the pro-proliferative gene *Ccnd1* that was previously recognized as a target of Wnt signaling [62]. This is compatible with the finding that proliferation defects observed in *Tbx2*-mutant lungs at E16.5 coincide with a strong decline of expression of this gene at this stage. However, the significance of downregulation of *Wnt2* and *Wnt5a* in the *Tbx2*-deficient lung remains unclear. We assume that it provides only a minor contribution to the observed changes.

At present, we cannot distinguish whether changes of Wnt signaling activity are secondary to cell cycle exit and/or upregulation of Cdkn1a and Cdkn1b or represent an independent branch of Tbx2 transcriptional activity in the lung mesenchyme. Unfortunately, the recovery of mice triple mutant for *Tbx2*, *Cdkn1a* and *Cdkn1b* for analysis of signaling pathways at E14.5 is extremely inefficient. The finding that constitutive expression of Tbx2 in the lung mesenchyme of adult mice did not increase canonical Wnt signaling, suggests that Tbx2 is not sufficient to activate this pathway. However, Tbx2 may be required for repression of an inhibitor of Wnt signaling to maintain this pathway during branching morphogenesis. The relevance of the control of canonical Wnt signaling by Tbx2 in the lung mesenchyme will be addressed in future experiments.

## Materials and Methods

### Ethics statement

All animal work conducted for this study was approved by H. Hedrich, state head of the animal facility at Medizinische Hochschule Hannover and performed according to German legislation.

### Mice and genonotyping

Mice carrying a null allele of *Cdkn1a* (*Cdkn1a^tm1Tyj^*, synonym *Cdkn1a^−^*) [63], a null allele of *Cdkn1b* (*Cdkn1b^tm1Mlf^*, synonym: *Cdkn1b^−^*) [64] or a null allele of *Tbx2* (*Tbx2^tm1.1(cre)Vmc^*, synonyms: *Tbx2^−^*, *Tbx2^cre^*) [21], and mice with integration of the human *TBX2* gene in the *Hprt* locus (*Hprt^tm2(CAG-TBX2,-EGFP)Akis^*, synonym: *Hprt^TBX2^*) [10] were maintained on an outbred (NMRI) background. For timed pregnancies, vaginal plugs were checked in the morning after mating; noon was taken as embryonic day (E) 0.5. Pregnant females were sacrificed by cervical dislocation; embryos were harvested in phosphate-buffered saline, decapitated, fixed in 4% paraformaldehyde overnight, and stored in 100% methanol at −20°C before further use. Genomic DNA prepared from yolk sacs or tail biopsies was used for genotyping by polymerase chain reaction (PCR). For primers and conditions see Table S2.

### Histological analysis and immunofluorescence

Embryos were embedded in paraffin and sectioned to 5 µm. For histological analyses, sections were stained with haematoxylin and eosin (HE), Masson's trichrome (Masson's) and picrosirius red (Sirius red) following standard protocols. For the detection of antigens, antigen retrieval was performed using citrate-based antigen unmasking solution (H-3300, Vector Laboratories Inc). Sections were pressure-cooked for 5 min and signal amplification was performed with the Tyramide Signal Amplification (TSA) system (NEL702001KT, Perkin Elmer LAS) or the DAB substrate kit (SK-4100, Vector Laboratories Inc). The following primary antibodies were used: rabbit anti-mouse E-cadherin (gift from Rolf Kemler, MPI for Immunobiology and Epigenetics, Freiburg/Germany) [65], rabbit polyclonal antibody against GFP (1∶200, sc-8334, Santa Cruz), mouse monoclonal antibody against GFP (1∶200, 11814460001, Roche), monoclonal antibody against alpha smooth muscle actin, Cy3-conjugate (1∶200, C 6198, Sigma), monoclonal antibody against alpha smooth muscle actin, FITC-conjugate (1∶200, F3777, Sigma), rabbit polyclonal against SM22a (transgelin, 1∶200, ab14106, Abcam), rat monoclonal antibody against endomucin (1∶2, gift from Dietmar Vestweber, MPI for Molecular Medicine, Münster/Germany) [66], rabbit polyclonal antibodies against Tbx2 (1∶100, ab33298, Abcam), Cdkn1a (1∶200, sc-397, SantaCruz), Cdkn1b (1∶200, 554069, BD Biosciences), uteroglobin (1∶200, ab40873, Abcam), cytokeratin14 (1∶200, ab7800, Abcam), Tubb4a (1∶100, ab11315, Abcam), prosurfactant protein C (1∶200, ab40879, Abcam), Sox2 (1∶100, ab97959, Abcam), Sox9 (1∶200, ab5535, Millipore), aquaporin5 (1∶100, ab92320, Abcam), hamster monoclonal against podoplanin (1∶50, ab11936, Abcam) and mouse monoclonal against BrdU (1∶100, 1170376, Roche). For immunofluorescent stainings on adult sections or double immunofluorescent stainings with two primary mouse antibodies the Biotinylated Mouse on Mouse (M.O.M.) Anti-Mouse Ig Reagent (Vector laboratories) was used.

### Organ culture

For analysis of branching morphogenesis E11.5 or E12.0 lung rudiments were dissected and kept on Transwell permeable 0.4-µm pore size, PET 6-well plates (Corning) supplied with DMEM supplemented with 10% fetal calf serum (Biowest), 2 mM Glutamax, 100 units/ml Penicillin, 100 µg/ml Streptomycin (Gibco). Lungs were cultivated at 37°C and 5% CO_2_ for 2 to 6 days and the number of branching endpoints was counted.

### Cell culture and siRNA

Human MCF-7 breast adenocarcinoma cell line was cultured in RPMI 1640 with Glutamax (Gibco) supplemented with 10% FBS, MEM non-essential amino acids (Gibco), 1 mM sodium pyruvate (Gibco), 10 µg/ml human insulin (Roche) and 100 units/ml Penicillin, 100 µg/ml Streptomycin (Gibco). Mouse B16 melanoma cells were cultured in RPMI 1640 with glutamax, supplemented with 10% FBS and 100 units/ml Penicillin, 100 µg/ml Streptomycin.

Downregulation of TBX2 or Tbx2 was achieved by siRNA exactly as recently described [12].

### *In situ* hybridization analysis

Whole-mount *in situ* hybridization was performed following a standard procedure with digoxigenin-labeled antisense riboprobes [67]. Stained specimens were transferred in 80% glycerol prior to documentation. *In situ* hybridization on 10 µm paraffin sections was done essentially as described [68]. For each marker at least three independent specimens were analyzed.

### Proliferation and apoptosis assays

Cell proliferation in embryonic and adult lungs was investigated by detection of incorporated 5-bromo-2′-deoxyuridine (BrdU) similar to published protocols [69]. At least nine sections from three individual embryos per genotype and stage were used for quantification. Statistical analysis was performed using the two-tailed Student's t-test. Data were expressed as mean ± standard deviation. Differences were considered significant when the P-value was below 0.05.

For detection of apoptotic cells in 5 µm paraffin sections of embryos, the terminal deoxynucleotidyl transferase-mediated nick-end labeling (TUNEL) assay was performed as recommended by the manufacturer (Serologicals Corp.) of the ApopTag kit used.

### Semi-quantitative reverse transcription PCR

Total RNA was extracted from dissected lungs with RNAPure reagent (Peqlab). RNA (500 ng) was reverse transcribed with RevertAid H Minus reverse transcriptase (Fermentas). For semiquantitative PCR, the number of cycles was adjusted to the mid-logarithmic phase. Quantification was performed with Quantity One software (Bio-Rad). Assays were performed at least twice in duplicate, and statistical analysis was done as previously described [9]. For primers and PCR conditions see Table S3.

### Chromatin immunoprecipitation (ChIP) assays

2ChIP was performed essentially as previously described [70]. Dissected E15.5 lung tissue was treated with 4% paraformaldehyde overnight. The DNA-containing supernatants were incubated overnight with anti-Tbx2 antibodies and collected on protein G beads. Cross-linked products were reversed by cooking for 15 min, treated with Proteinase K and RNAse H at 56°C for 30 min and the immunoprecipitated DNA was purified. Primers for PCR amplification were 5′-CCGAGAGGTGTGAGCCGC-3′ (Cdkn1a-f1) and 5′- GTCATCCACCTGCCGCGG-3′ (Cdkn1a-r1); 5′-GGCTTAGATTCCCAGAGGG-3′ (Cdkn1af2) and 5′-TTCTGGGGACACCCACTGG-3′ (Cdkn1a-r2) for the *Cdkn1a* promoter and 5′- CAAGTTCAGTAAACTAAGTAGG-3′ (Cdkn1b-f1) and 5′- GCACATATGTGGACAAACTCG-3′ (Cdkn1b-r1) for the 5′-T-site in the *Cdkn1b* promoter. For the intron located T-site 5′-ATATACCTTCTACAGACATAGC-3′ (Cdkn1b-f2) and 5′- GCTTTTGACTAGAGTCTTATGG-3′ (Cdkn1b-r2) primers were used. Primers for the negative control region were 5′-CTCTGAAACTCGAACAGGCC-3′ (ncr-f1) and 5′- ACTCTGAATTGGATTCCTAGC-3′ (ncr-r1).

### Image analysis

Sections were photographed using a Leica DM5000 microscope with a Leica DFC300FX digital camera. Whole mount specimens were photographed on a Leica M420 microscope with a Fujix digital camera HC-300Z. Images were processed in Adobe Photoshop CS3.

## Supporting Information

Figure S1 *Tbx2* is required for lung epithelial branching. (A) Morphology of lung explants from E12.0 wildtype and *Tbx2^−/−^* embryos at the start and after 6 days of culture. Boxes show regions that were magnified to see branching endpoints in the lower panel. (B) Quantitative and statistical analysis of branching morphogenesis of E12.0 lung rudiments cultured for 0, 2, 4 and 6 days by counting of peripheral branching endpoints. Branching endpoints were not significantly (ns) reduced after four days of culture (p = 0.08). After 6 days of culture branching endpoints were highly significantly reduced from 116+/−9 in wildtype to 70+/−2 in *Tbx2*-deficient cultures (p = 1×10^−4^). Scale bars represent 500 µm. For statistics see Table S1G.
(TIF)

Figure S2 Determination of proliferation in the epithelium of *Tbx2*-deficient lungs. (Co-) immunofluorescence analysis of BrdU and the distal epithelial marker Sox2 (A,C) and the proximal marker Sox9 (B,D) in sections of E14.5 and E16.5 wildtype and *Tbx2*-deficient (*Tbx2^−/−^*) lungs. Reduced proliferation was found in the distal but not in the proximal region of the lung epithelium. Scale bars represent 50 µm.
(TIF)

Figure S3 S100a4-positive fibroblasts are highly proliferative. BrdU incorporation assay analyzed by immunofluorescence on E14.5 lung sections co-stained for S100a4 shows that all S100a4-positive fibroblasts proliferate. Arrowheads mark S100a4-positive cells. Scale bars represent 50 µm.
(TIF)

Figure S4 Minor changes of epithelial differentiation in *Tbx2*-deficient lungs. Immunohistochemistry on frontal section of wildtype (wt) and *Tbx2-*deficient (*Tbx2^−/−^*) lungs for regionalization of proximal (Sox2) and distal airways (Sox9), for differentiation of proximal airway epithelium into tracheal basal cells (Krt14), ciliated cells (Tubb4a) and Clara cells (Scgb1a1), and for differentiation of alveolar epithelial cells type 1 (Pdpn, Aqp5) and type 2 (Sftpc1). Genotypes, probes and stages are as indicated. All markers are appropriately activated in the *Tbx2*-deficient lung epithelium. Pdpn and Aqp5, however, are not maintained at the appropriate levels. Scale bars represent 100 µm.
(TIF)

Figure S5 *Cdkn1a* and *Cdkn1b* loci harbor binding sites for T-box proteins. Schemes depicting the genomic organization of the *Cdkn1a* and the *Cdkn1b* locus. Exon coding sequences are indicated as black boxes, white boxes refer to untranslated sequences within exons. Arrows mark positions of primers used to amplify DNA fragments for ChIP analysis. Nucleotide sequences refer to conserved binding sites for T-box proteins. Black numbers refer to exons, red numbers indicate the size of the genomic fragments in bp.
(TIF)

Figure S6 Endogenous Tbx2/TBX2 represses endogenous Cdkn1b in B16 melanoma and MCF-7 breast cancer cell lines. Immunofluorescent stainings of TBX2/Tbx2 and CDKN1B/Cdkn1b protein in human MCF-7 (human) and mouse B16 cell lines transfected with siRNA specific for TBX2/Tbx2 or a non-silencing control siRNA. Knock-down of TBX2/Tbx2 results in upregulation of CDKN1B/Cdkn1b expression in both cell lines three days after treatment. Scale bars represent 50 µm.
(TIF)

Figure S7 Quantification of Tbx2/TBX2 protein in TBX2-overexpressing lungs by Western blot analysis. 4 lungs each of E18.5 control and *Tbx2^cre/+^*;*Hprt^TBX2/+^* embryos were pooled and lysed in 1 ml of Nonidet-P40 buffer. After sonification, 2 µl of the lysate (1∶500) and 0.2 µl (1∶5000), respectively, were loaded on the gel and analyzed with an anti-Tbx2 antibody after blotting. Levels of Tbx2/TBX2 protein were comparable between samples indicating that TBX2 misexpression occurs within physiological range.
(TIF)

Figure S8 Branching morphogenesis is not affected in explant cultures of *Tbx2^cre/+^*;*Hprt^TBX2/+^* lungs. (A) Morphology of lung explants from E12.0 wildtype and *Tbx2^cre/+^*;*Hprt^TBX2/+^* embryos at the start and after 6 days of culture. Boxes show regions that were magnified to see branching endpoints in the lower panel. (B) Quantitative and statistical analysis of branching morphogenesis of E12.0 lung rudiments cultured for 0, 2, 4 and 6 days by counting of peripheral branching endpoints does not detect differences in branching morphogenesis between wildtype and *Tbx2^cre/+^*;*Hprt^TBX2/+^*embryos. Scale bars represent 500 µm. For statistics see Table S1H.
(TIF)

Figure S9 Normal epithelial differentiation in P40 *Tbx2^cre/+^*;*Hprt^TBX2/+^* mice. Immunofluorescence (Tagln) and immunohistochemistry (Emcn, Sox2, Sox9, Tubb4a, Scgb1a1, Aqp5, Pdpn, Sftpc1) on frontal section of wildtype (wt) and *TBX2-*overexpressing (*Tbx2^cre/+^*;*Hprt^TBX2/+^*) lungs for endothelial cells (Emcn), for smooth muscle cells surrounding the proximal airways (Tagln), for regionalization of proximal airways (Sox2) and distal airways (Sox9), for differentiation of proximal airway epithelium into ciliated cells (Tubb4a) and Clara cells (Scgb1a1), and for differentiation of alveolar epithelial cells type 1 (Aqp5, Pdpn) and type 2 (Sftpc1). Genotypes, probes and stages are as indicated. All markers are appropriately expressed in *Tbx2^cre/+^*;*Hprt^TBX2/+^* mice. Scale bars represent 100 µm.
(TIF)

Figure S10 Loss of *Cdkn1a* and *Cdkn1b* increases lung growth. Analysis of wildtype, *Cdkn1a^−/−^*;*Cdkn1b^−/−^* and *Cdkn1a^−/−^*;*Cdkn1b^+/−^* mice at P40. (A) Morphology of P40 mice and lungs. (B) Statistical analysis of relative body and lung weight. Relative body weight is increased by 20% both in *Cdkn1a^−/−^*;*Cdkn1b^−/−^ and Cdkn1a^−/−^*;*Cdkn1b^+/−^* mice; the lung weight (relative to the body weight) is increased in *Cdkn1a^−/−^*;*Cdkn1b^−/−^* 1.35-fold (n = 2). (C) Histological analysis by haematoxylin and eosin (HE), Sirius red and Masson's staining on sections of P40 lungs. (D) BrdU incorporation assay of frontal sections of the lung. *Cdkn1a*/*Cdkn1b* double-mutant mice show increased BrdU incorporation. (E) Analysis of apoptosis by TUNEL staining. (F) Immunofluorescence analysis of Fn1, Postn, S100a4, Cdkn1a and Cdkn1b expression on lung sections at P40. Genotypes are as indicated. Scale bars in A represent 1 cm and 2.5 mm, respectively. Scale bars in C,D,E,F represent 50 µm. For statistics see Table S1I.
(TIF)

Figure S11 ChIP-Seq analysis of Tbx3-binding to *Cdkn1*a and *Cdkn1b* loci in atrial cardiomyocytes as identified in [53]. Graphical representation of binding peaks (in red) of the transcriptional repressor Tbx3 to the genomic region of *Cdkn1a* (A) and *Cdkn1b* (B). Boxes show genomic regions that are subsequently represented in a magnified fashion. Scale bars are indicated. Arrows refer to primers used to amplify genomic fragments that harbor TBEs (see Figure 6).
(TIF)

Table S1 Statistical analyses of morphological, cellular and molecular changes in lungs with loss or gain of Tbx2 activity.
(PDF)

Table S2 Primers and conditions of PCRs for genotyping of mouse strains.
(PDF)

Table S3 Primers and conditions for analysis of expression by semi-quantitative RT-PCR.
(PDF)
