# Supplementary figures and images for: Tbx2 Controls Lung Growth by Direct Repression of the Cell Cycle Inhibitor Genes Cdkn1a and Cdkn1b
Source: PLoS Genet. 2013 Jan 17;9(1):e1003189. doi: 10.1371/journal.pgen.1003189 (PMC3547831; doi:10.1371/journal.pgen.1003189)

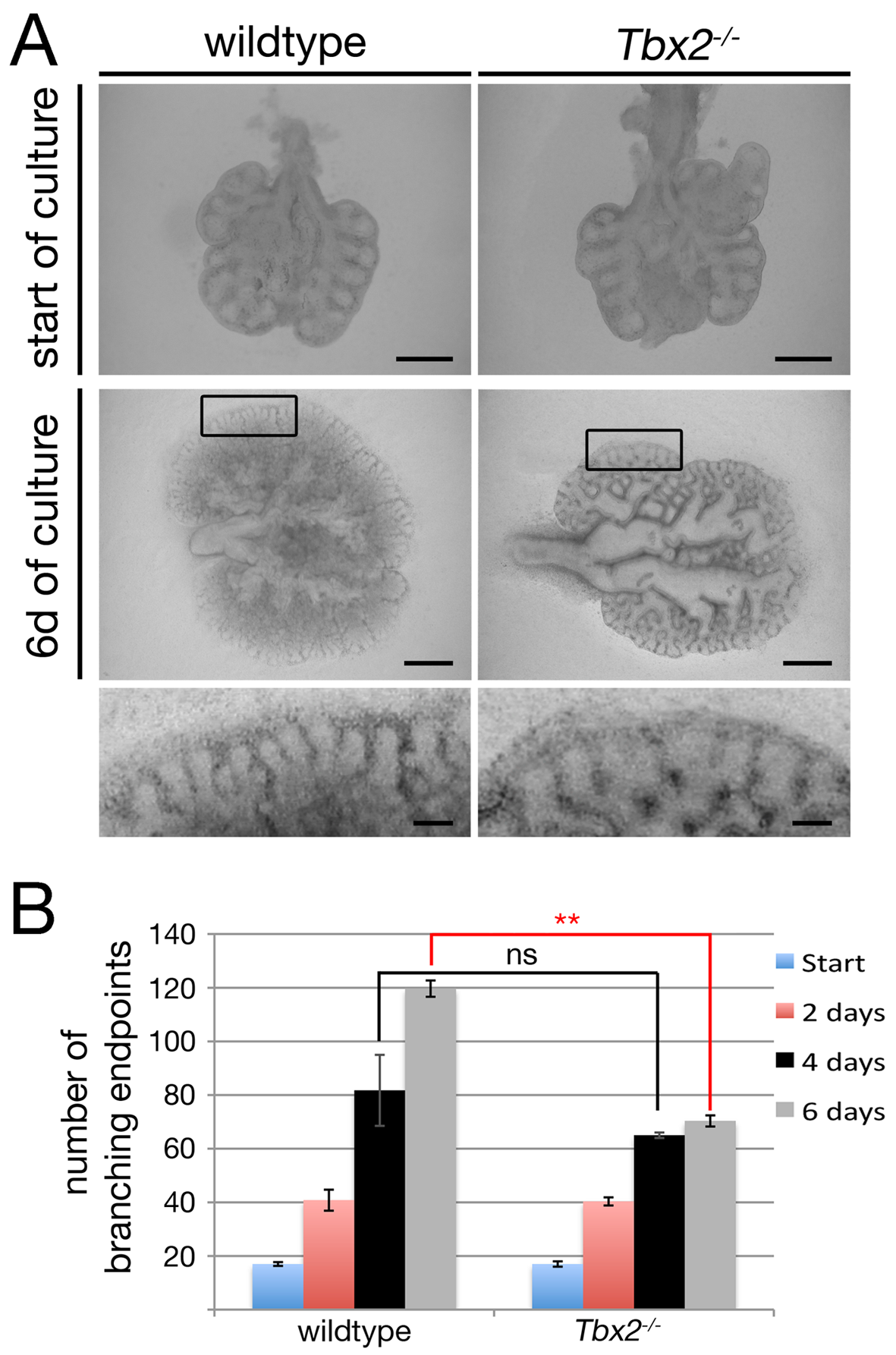

Supplement: Figure S1 — Tbx2 is required for lung epithelial branching. (A) Morphology of lung explants from E12.0 wildtype and Tbx2−/− embryos at the start and after 6 days of culture. Boxes show regions that were magnified to see branching endpoints in the lower panel. (B) Quantitative and statistical analysis of branching morphogenesis of E12.0 lung rudiments cultured for 0, 2, 4 and 6 days by counting of peripheral branching endpoints. Branching endpoints were not significantly (ns) reduced after four days of culture (p = 0.08). After 6 days of culture branching endpoints were highly significantly reduced from 116+/−9 in wildtype to 70+/−2 in Tbx2-deficient cultures (p = 1×10^−4^). Scale bars represent 500 µm. For statistics see Table S1G. (TIF) [file pgen.1003189.s001.tif]

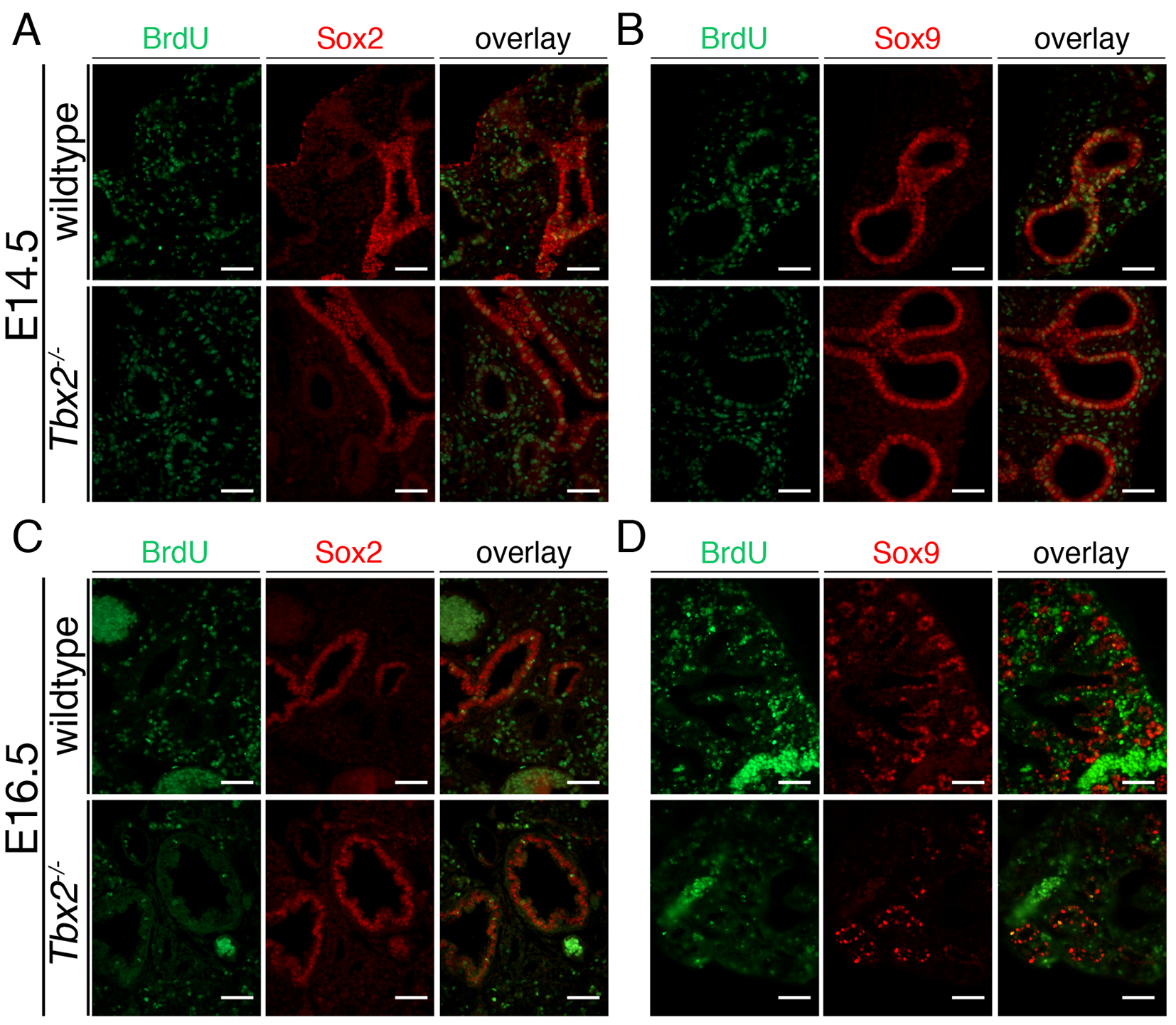

Supplement: Figure S2 — Determination of proliferation in the epithelium of Tbx2-deficient lungs. (Co-) immunofluorescence analysis of BrdU and the distal epithelial marker Sox2 (A,C) and the proximal marker Sox9 (B,D) in sections of E14.5 and E16.5 wildtype and Tbx2-deficient (Tbx2−/−) lungs. Reduced proliferation was found in the distal but not in the proximal region of the lung epithelium. Scale bars represent 50 µm. (TIF) [file pgen.1003189.s002.tif]

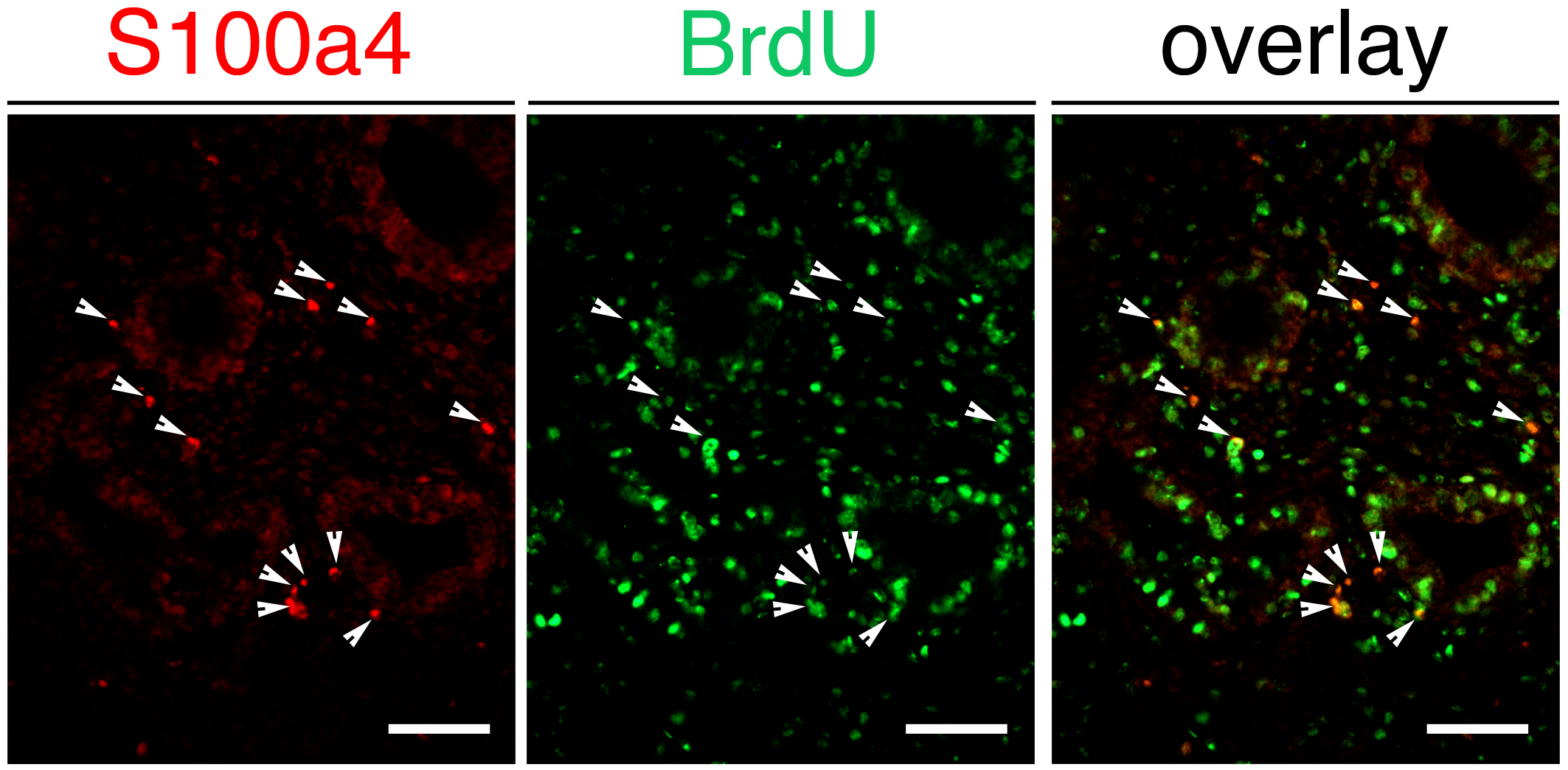

Supplement: Figure S3 — S100a4-positive fibroblasts are highly proliferative. BrdU incorporation assay analyzed by immunofluorescence on E14.5 lung sections co-stained for S100a4 shows that all S100a4-positive fibroblasts proliferate. Arrowheads mark S100a4-positive cells. Scale bars represent 50 µm. (TIF) [file pgen.1003189.s003.tif]

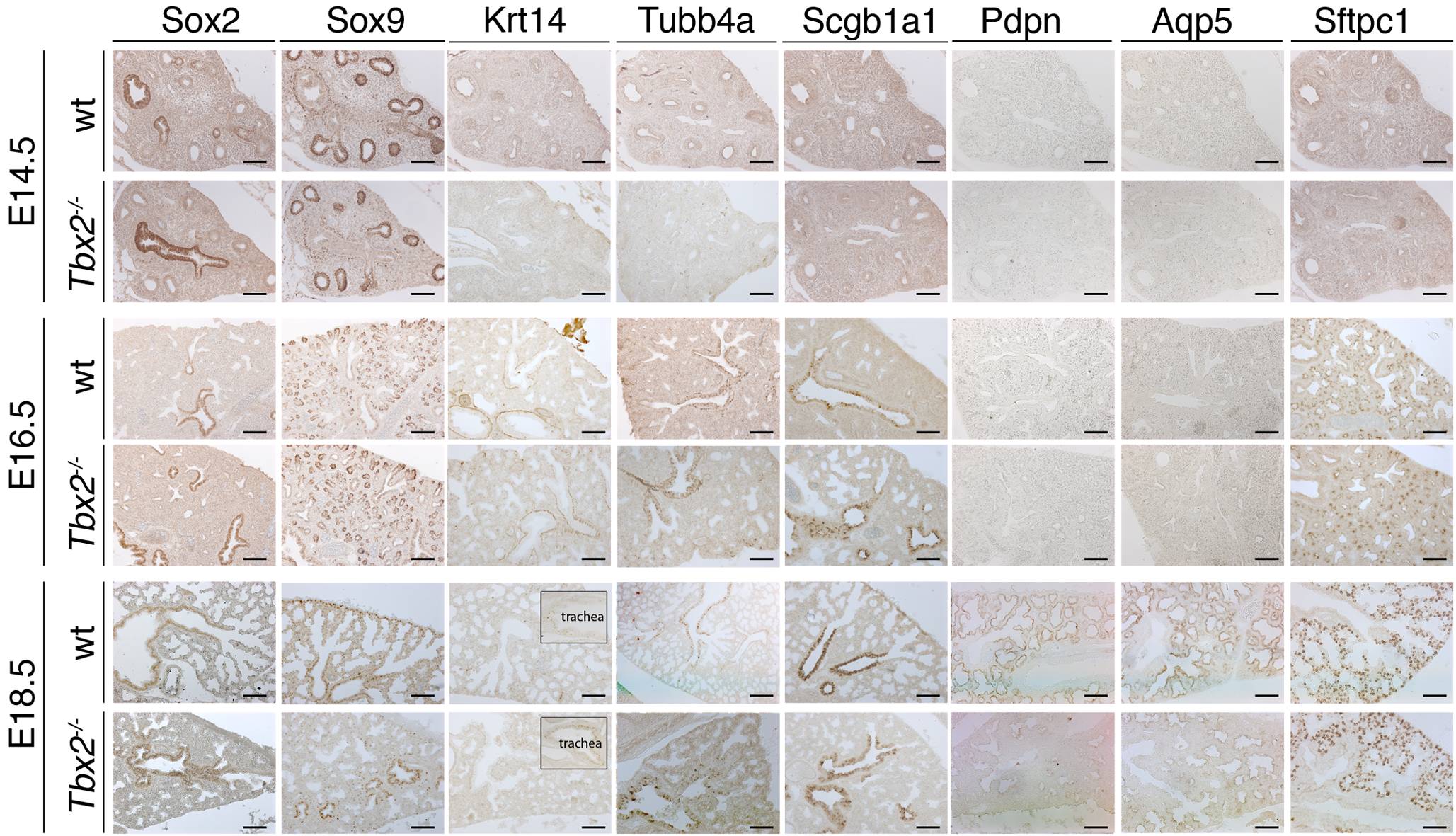

Supplement: Figure S4 — Minor changes of epithelial differentiation in Tbx2-deficient lungs. Immunohistochemistry on frontal section of wildtype (wt) and Tbx2-deficient (Tbx2−/−) lungs for regionalization of proximal (Sox2) and distal airways (Sox9), for differentiation of proximal airway epithelium into tracheal basal cells (Krt14), ciliated cells (Tubb4a) and Clara cells (Scgb1a1), and for differentiation of alveolar epithelial cells type 1 (Pdpn, Aqp5) and type 2 (Sftpc1). Genotypes, probes and stages are as indicated. All markers are appropriately activated in the Tbx2-deficient lung epithelium. Pdpn and Aqp5, however, are not maintained at the appropriate levels. Scale bars represent 100 µm. (TIF) [file pgen.1003189.s004.tif]

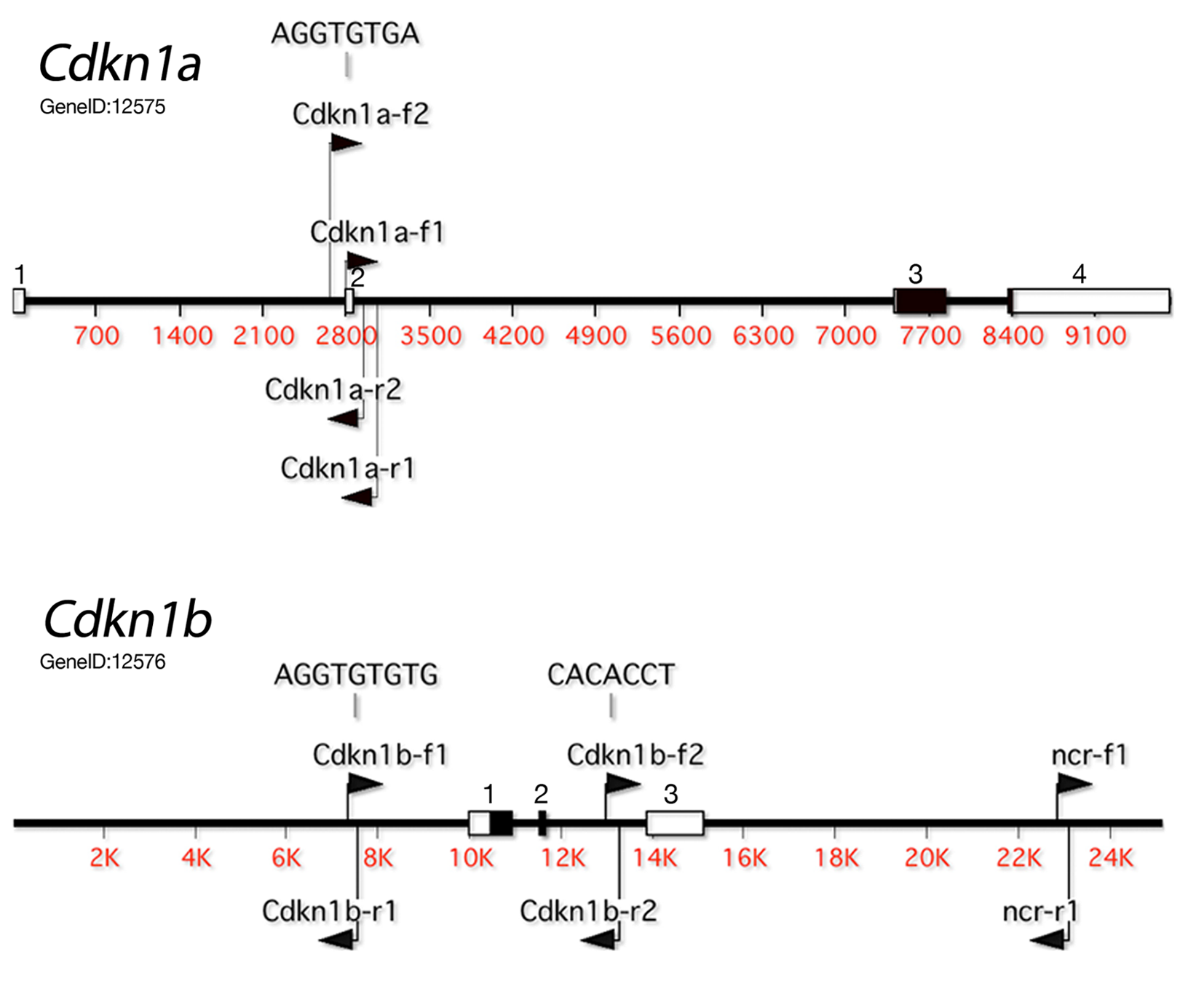

Supplement: Figure S5 — Cdkn1a and Cdkn1b loci harbor binding sites for T-box proteins. Schemes depicting the genomic organization of the Cdkn1a and the Cdkn1b locus. Exon coding sequences are indicated as black boxes, white boxes refer to untranslated sequences within exons. Arrows mark positions of primers used to amplify DNA fragments for ChIP analysis. Nucleotide sequences refer to conserved binding sites for T-box proteins. Black numbers refer to exons, red numbers indicate the size of the genomic fragments in bp. (TIF) [file pgen.1003189.s005.tif]

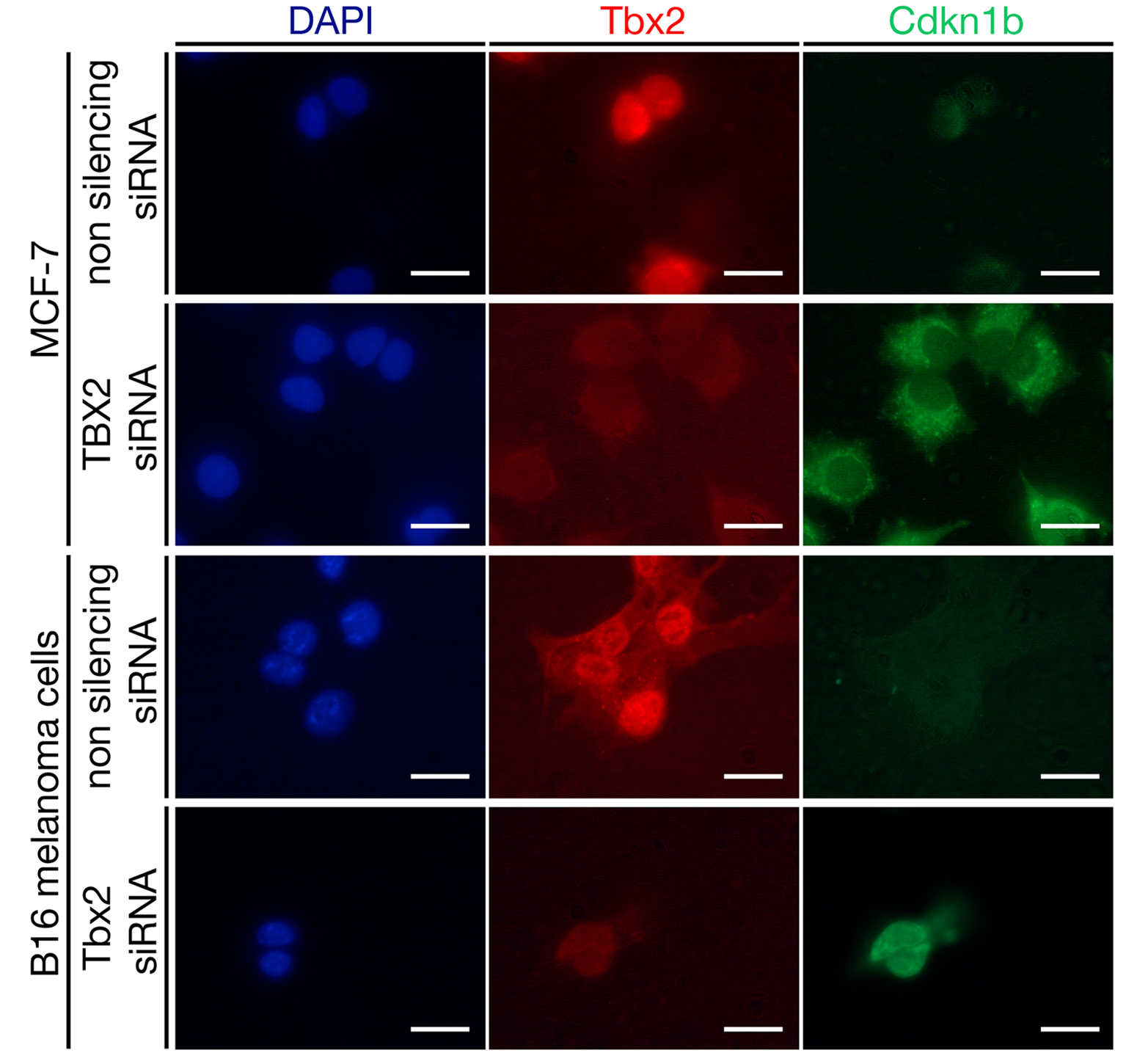

Supplement: Figure S6 — Endogenous Tbx2/TBX2 represses endogenous Cdkn1b in B16 melanoma and MCF-7 breast cancer cell lines. Immunofluorescent stainings of TBX2/Tbx2 and CDKN1B/Cdkn1b protein in human MCF-7 (human) and mouse B16 cell lines transfected with siRNA specific for TBX2/Tbx2 or a non-silencing control siRNA. Knock-down of TBX2/Tbx2 results in upregulation of CDKN1B/Cdkn1b expression in both cell lines three days after treatment. Scale bars represent 50 µm. (TIF) [file pgen.1003189.s006.tif]

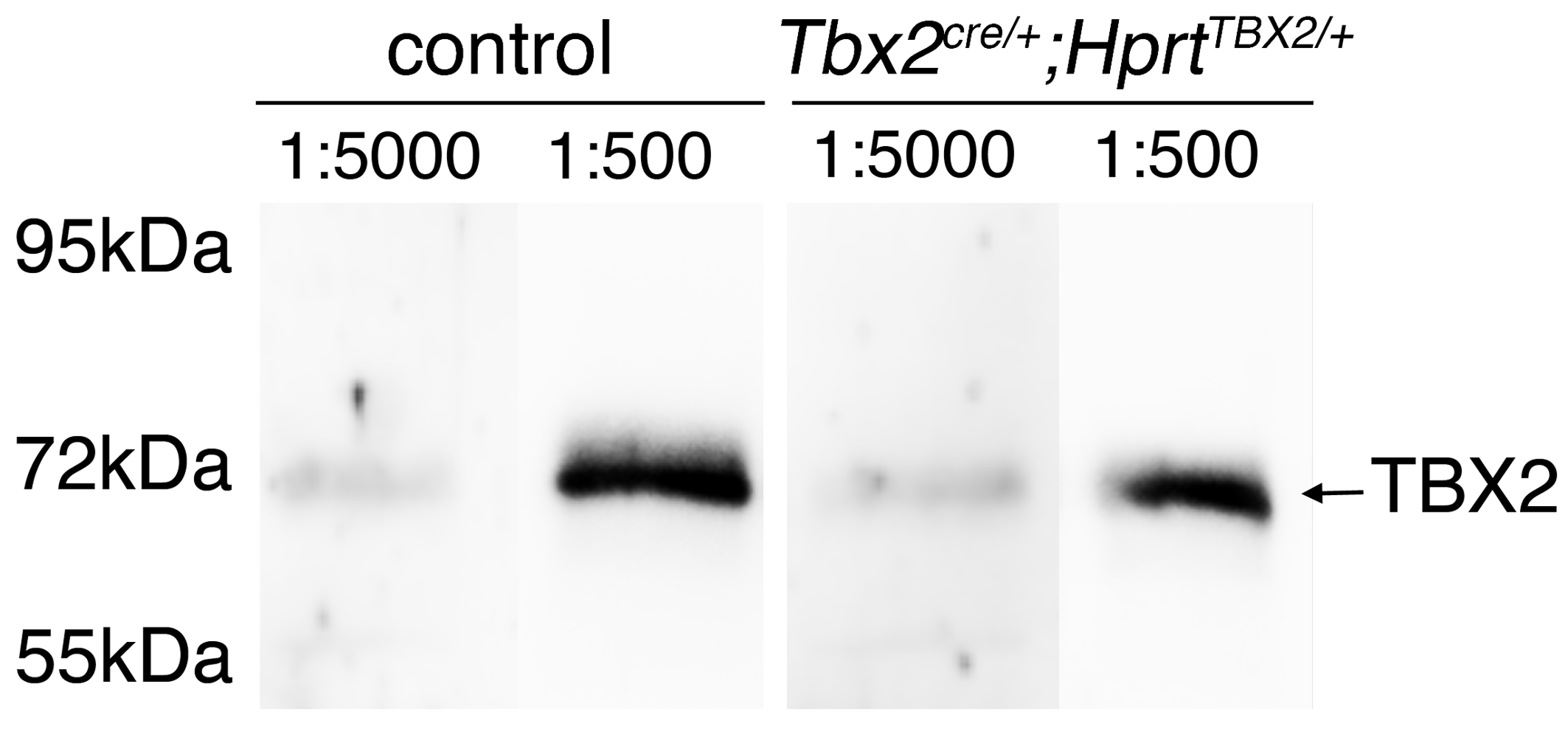

Supplement: Figure S7 — Quantification of Tbx2/TBX2 protein in TBX2-overexpressing lungs by Western blot analysis. 4 lungs each of E18.5 control and Tbx2cre/+;HprtTBX2/+ embryos were pooled and lysed in 1 ml of Nonidet-P40 buffer. After sonification, 2 µl of the lysate (1∶500) and 0.2 µl (1∶5000), respectively, were loaded on the gel and analyzed with an anti-Tbx2 antibody after blotting. Levels of Tbx2/TBX2 protein were comparable between samples indicating that TBX2 misexpression occurs within physiological range. (TIF) [file pgen.1003189.s007.tif]

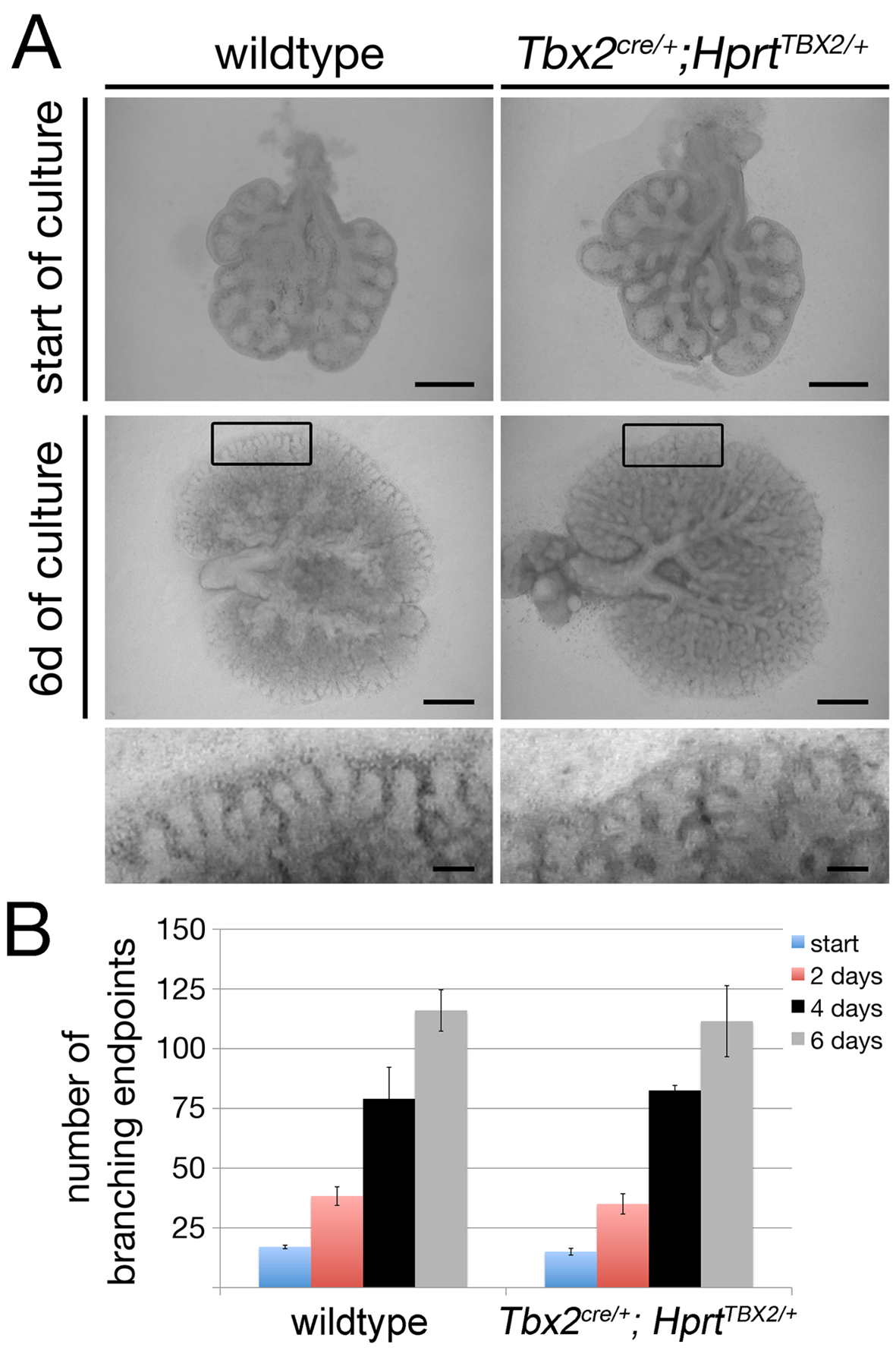

Supplement: Figure S8 — Branching morphogenesis is not affected in explant cultures of Tbx2cre/+;HprtTBX2/+ lungs. (A) Morphology of lung explants from E12.0 wildtype and Tbx2cre/+;HprtTBX2/+ embryos at the start and after 6 days of culture. Boxes show regions that were magnified to see branching endpoints in the lower panel. (B) Quantitative and statistical analysis of branching morphogenesis of E12.0 lung rudiments cultured for 0, 2, 4 and 6 days by counting of peripheral branching endpoints does not detect differences in branching morphogenesis between wildtype and Tbx2cre/+;HprtTBX2/+embryos. Scale bars represent 500 µm. For statistics see Table S1H. (TIF) [file pgen.1003189.s008.tif]

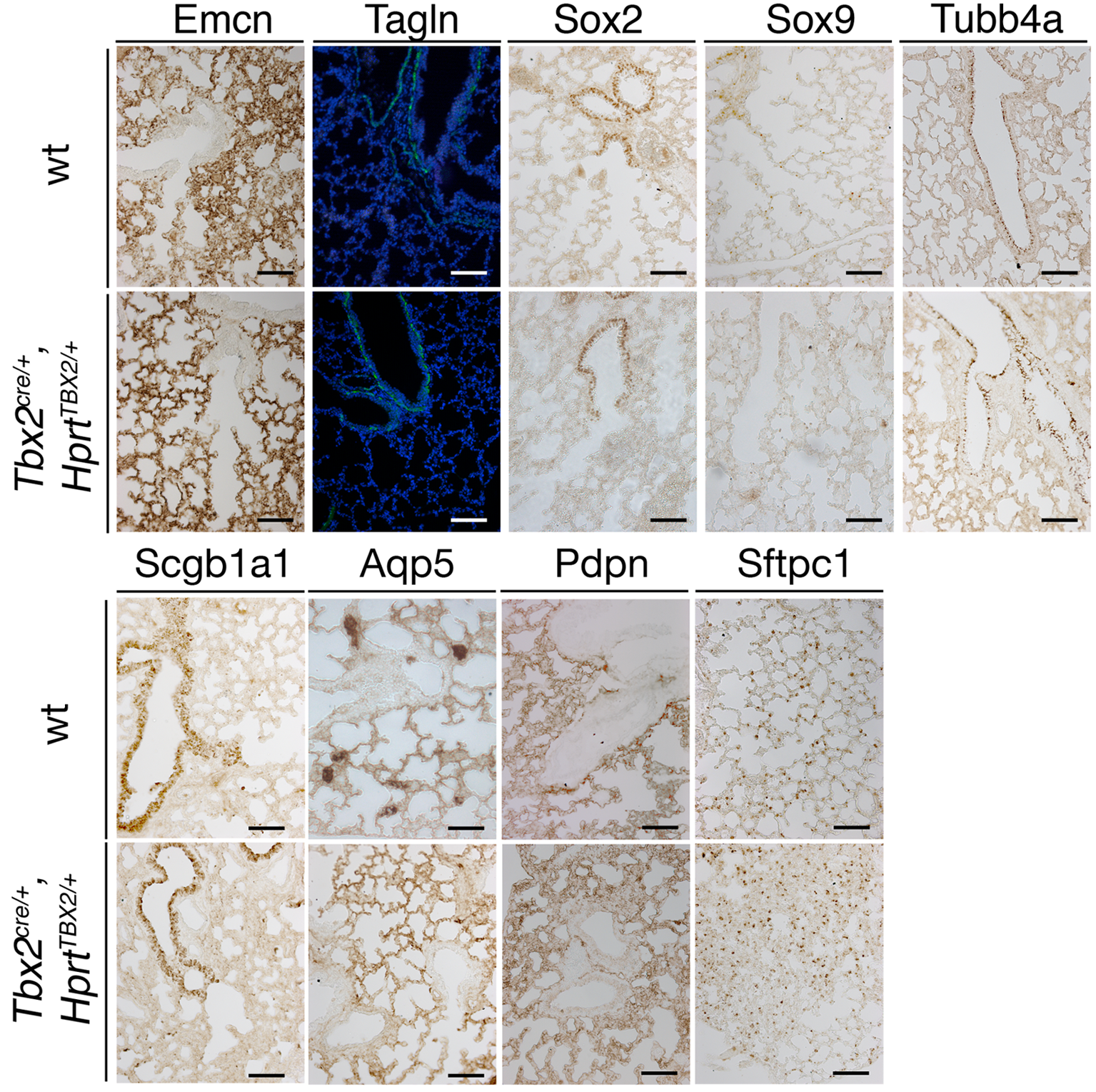

Supplement: Figure S9 — Normal epithelial differentiation in P40 Tbx2cre/+;HprtTBX2/+ mice. Immunofluorescence (Tagln) and immunohistochemistry (Emcn, Sox2, Sox9, Tubb4a, Scgb1a1, Aqp5, Pdpn, Sftpc1) on frontal section of wildtype (wt) and TBX2-overexpressing (Tbx2cre/+;HprtTBX2/+) lungs for endothelial cells (Emcn), for smooth muscle cells surrounding the proximal airways (Tagln), for regionalization of proximal airways (Sox2) and distal airways (Sox9), for differentiation of proximal airway epithelium into ciliated cells (Tubb4a) and Clara cells (Scgb1a1), and for differentiation of alveolar epithelial cells type 1 (Aqp5, Pdpn) and type 2 (Sftpc1). Genotypes, probes and stages are as indicated. All markers are appropriately expressed in Tbx2cre/+;HprtTBX2/+ mice. Scale bars represent 100 µm. (TIF) [file pgen.1003189.s009.tif]

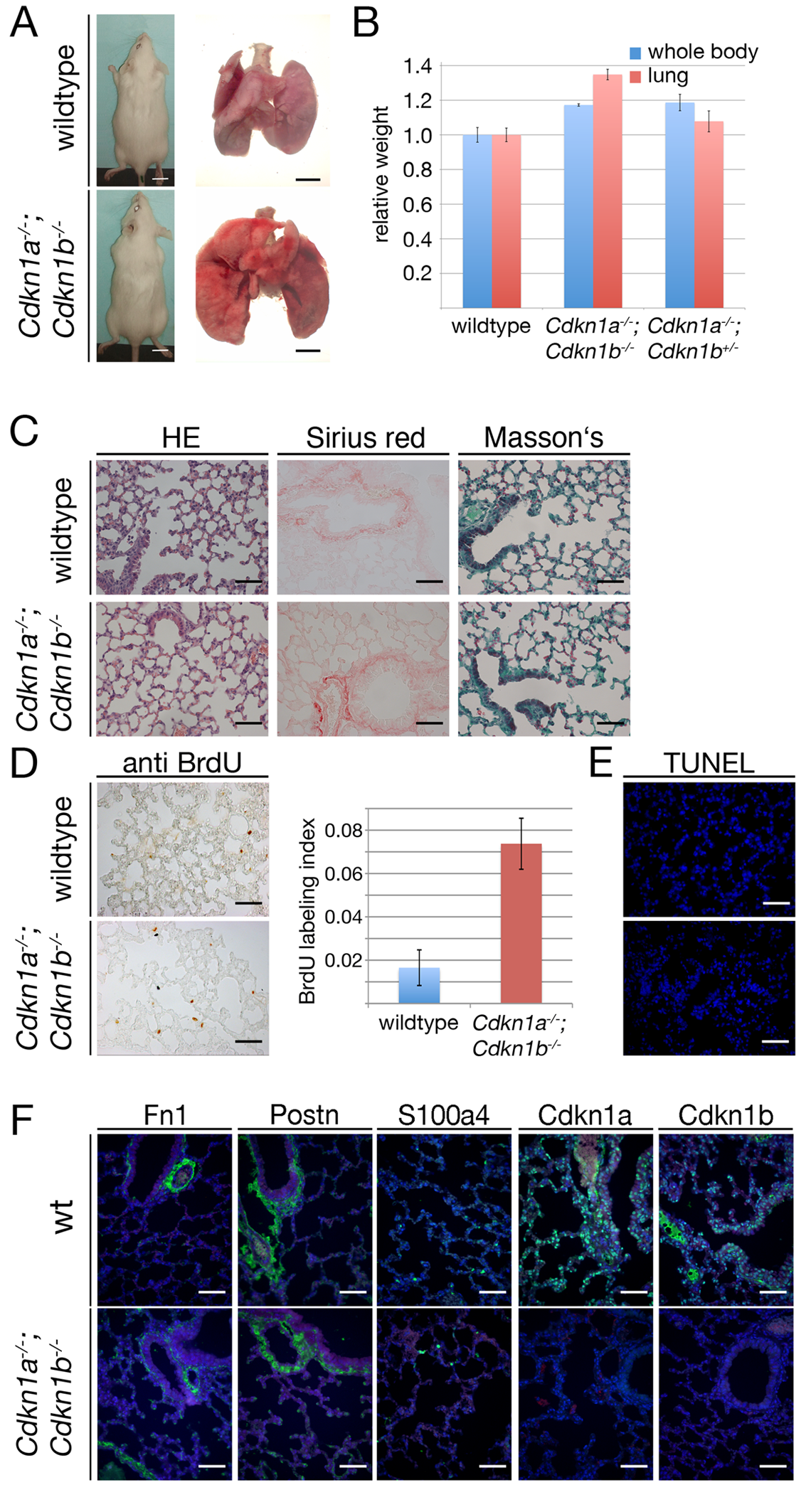

Supplement: Figure S10 — Loss of Cdkn1a and Cdkn1b increases lung growth. Analysis of wildtype, Cdkn1a−/−;Cdkn1b−/− and Cdkn1a−/−;Cdkn1b+/− mice at P40. (A) Morphology of P40 mice and lungs. (B) Statistical analysis of relative body and lung weight. Relative body weight is increased by 20% both in Cdkn1a−/−;Cdkn1b−/− and Cdkn1a−/−;Cdkn1b+/− mice; the lung weight (relative to the body weight) is increased in Cdkn1a−/−;Cdkn1b−/− 1.35-fold (n = 2). (C) Histological analysis by haematoxylin and eosin (HE), Sirius red and Masson's staining on sections of P40 lungs. (D) BrdU incorporation assay of frontal sections of the lung. Cdkn1a/Cdkn1b double-mutant mice show increased BrdU incorporation. (E) Analysis of apoptosis by TUNEL staining. (F) Immunofluorescence analysis of Fn1, Postn, S100a4, Cdkn1a and Cdkn1b expression on lung sections at P40. Genotypes are as indicated. Scale bars in A represent 1 cm and 2.5 mm, respectively. Scale bars in C,D,E,F represent 50 µm. For statistics see Table S1I. (TIF) [file pgen.1003189.s010.tif]

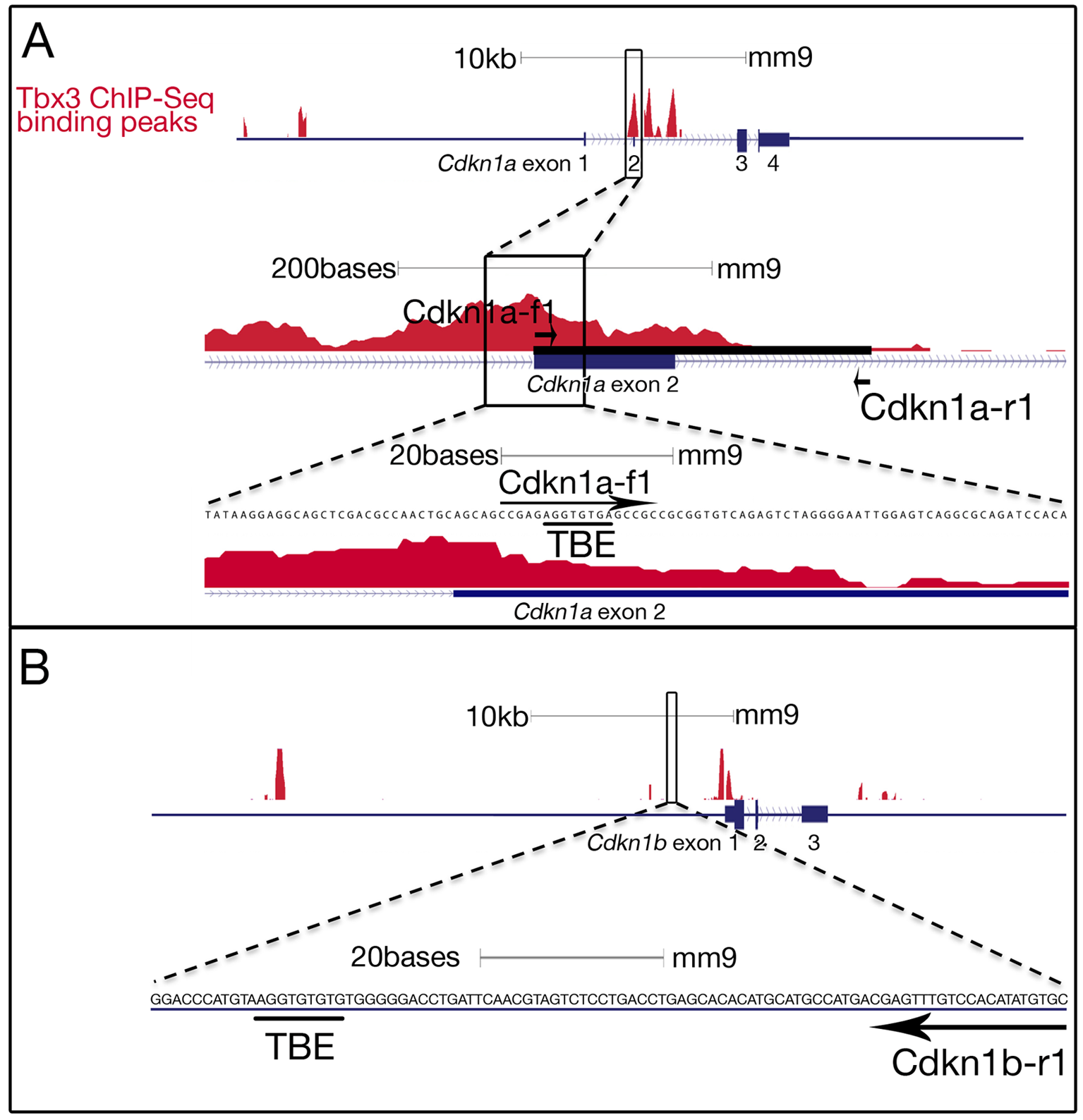

Supplement: Figure S11 — ChIP-Seq analysis of Tbx3-binding to Cdkn1a and Cdkn1b loci in atrial cardiomyocytes as identified in [53]. Graphical representation of binding peaks (in red) of the transcriptional repressor Tbx3 to the genomic region of Cdkn1a (A) and Cdkn1b (B). Boxes show genomic regions that are subsequently represented in a magnified fashion. Scale bars are indicated. Arrows refer to primers used to amplify genomic fragments that harbor TBEs (see Figure 6). (TIF) [file pgen.1003189.s011.tif]
