# Supplementary material for: Tbx2 Controls Lung Growth by Direct Repression of the Cell Cycle Inhibitor Genes Cdkn1a and Cdkn1b
Source: PLoS Genet. 2013 Jan 17;9(1):e1003189. doi: 10.1371/journal.pgen.1003189 (PMC3547831; doi:10.1371/journal.pgen.1003189)
Supplement: Table S1 — Statistical analyses of morphological, cellular and molecular changes in lungs with loss or gain of Tbx2 activity. (PDF) [file pgen.1003189.s012.pdf]

A

relative organ weight of wildtype and $Tbx2^{cre/cre}$ mice referring to figure 1

E18.5	relative liver weight (wt set to 1)	standard deviation	P-value wt vs. $Tbx2^{cre/+}$, $Hprt^{TBX2/+}$	n	relative lung weight (wt set to 1)	standard deviation	P-value wt vs. $Tbx2^{cre/+}$, $Hprt^{TBX2/+}$	n
wildtype	1.0	0.0		3	1.000	0.016		3
$Tbx2^{cre/cre}$	1.1	0.0	p=0.0116	3	0.551	0.017	p<0.0005	3

E16.5	relative liver weight (wt set to 1)	standard deviation	P-value wt vs. $Tbx2^{cre/+}$, $Hprt^{TBX2/+}$	n	relative lung weight (wt set to 1)	standard deviation	P-value wt vs. $Tbx2^{cre/+}$, $Hprt^{TBX2/+}$	n
wildtype	1.00	0.01		4	1.00	0.09		4
$Tbx2^{cre/cre}$	0.91	0.08	0.074	4	0.33	0.18	p<0.0005	4

E14.5	relative liver weight (wt set to 1)	standard deviation	P-value wt vs. $Tbx2^{cre/+}$, $Hprt^{TBX2/+}$	n	relative lung weight (wt set to 1)	standard deviation	P-value wt vs. $Tbx2^{cre/+}$, $Hprt^{TBX2/+}$	n
wildtype	1.00	0.08		9	1.00	0.07		9
$Tbx2^{cre/cre}$	0.93	0.09	0.396	9	0.85	0.12	0.107	9

B

BrdU labeling indices referring to figure 2C

E14.5	average mesenchyme	standard deviation mesenchyme	P-value wt vs $Tbx2^{cre/cre}$ mesenchyme	n	average proximal epithelium	standard deviation prox. epithelium	P-value wt vs $Tbx2^{cre/cre}$ prox. epithelium	n	average distal epithelium	standard deviation distal epithelium	P-value wt vs $Tbx2^{cre/cre}$ distal epithelium	n	average diaphragm	standard deviation diaphragm	P-value wt vs $Tbx2^{cre/cre}$ diaphragm	n
wildtype	0.480	0.067		3	0.598	0.083		3	0.525	0.015		5	0.395	0.050		3
$Tbx2^{cre/cre}$	0.485	0.110	0.956	5	0.629	0.094	0.689	3	0.505	0.019	0.081	6	0.342	0.110	0.451	3

BrdU labeling indices referring to figure 2F

E16.5	average mesenchyme	standard deviation mesenchyme	P-value wt vs $Tbx2^{cre/cre}$ mesenchyme	n	average proximal epithelium	standard deviation prox. epithelium	P-value wt vs $Tbx2^{cre/cre}$ prox. epithelium	n	average distal epithelium	standard deviation distal epithelium	P-value wt vs $Tbx2^{cre/cre}$ distal epithelium	n	average diaphragm	standard deviation diaphragm	P-value wt vs $Tbx2^{cre/cre}$ diaphragm	n
wildtype	0.296	0.015		3	0.161	0.006		3	0.197	0.016		5	0.169	0.049		3
$Tbx2^{cre/cre}$	0.160	0.034	0.003	4	0.165	0.044	0.875	3	0.142	0.047	0.034	6	0.142	0.023	0.526	3

BrdU labeling indices referring to figure 2I

E18.5	average	standard deviation	P-value wt vs $Tbx2^{cre/cre}$	n
wildtype	0.055	0.007		4
$Tbx2^{cre/cre}$	0.066	0.010	0.485	4

C

statistics of RT-PCRs relative to wildtype referring to figure 5A

	Bmp2	*Bmp4*	*Bmpr1a*	*Bmpr2*	*Msx1*	*Fgf10*	*Fgfr2*	*Etv4*	*Shh*	*Ptch1*	*Wnt2*	*Wnt5a*	*Axin2*
E14.5 $Tbx2^{cre/cre}$	1.07	0.90	1.07	1.06	0.98	1.11	1.13	0.97	1.08	1.19	0.89	0.67	0.29
E16.5 $Tbx2^{cre/cre}$	0.92	0.52	1.08	0.49	0.60	1.04	1.06	1.17	0.33	0.82	0.41	0.65	0.46
E14.5 standard deviation $Tbx2^{cre/cre}$	0.07	0.12	0.14	0.13	0.14	0.13	0.21	0.07	0.10	0.33	0.30	0.12	0.11
E16.5 standard deviation $Tbx2^{cre/cre}$	0.05	0.04	0.07	0.08	0.12	0.16	0.13	0.07	0.12	0.19	0.08	0.03	0.14

statistics of RT-PCRs relative to wildtype referring to figure 5C

	Ccnd1	*Ccnd2*	*Ccnd3*	*Cdk1*	*Cdkn2a*	*Cdkn2a/Arf*	*Cdkn1a*	*Cdkn1b*	*Cdkn1c*	*Tbx3*
E14.5 $Tbx2^{cre/cre}$	1.25	1.26	1.17	1.15	1.01	1.17	3.60	4.23	1.05	0.93
E16.5 $Tbx2^{cre/cre}$	0.33	1.00	1.24	0.66	1.41	1.01	1.08	7.20	0.95	0.85
E14.5 standard deviation $Tbx2^{cre/cre}$	0.31	0.17	0.24	0.30	0.06	0.17	0.28	0.49	0.25	0.11
E16.5 standard deviation $Tbx2^{cre/cre}$	0.15	0.11	0.34	0.06	0.17	0.22	0.04	0.82	0.25	0.26

D

relative lung weights of E18.5 lungs of compound mutants of *Tbx2, Cdkn1a* and *Cdkn1b* referring to figure 6B

	relative lung weight	standard deviation	n
$Tbx2^{cre/+}$	1	0.02	7
$Tbx2^{cre/cre}$	0.55	0.02	3
$Tbx2^{cre/cre};Cdkn1a^{-/-}$	0.38	0.13	5
$Tbx2^{cre/cre};Cdkn1b^{-/-}$	0.42	0.12	4
$Tbx2^{cre/cre};Cdkn1a^{-/-}$; $Cdkn1b^{-/-}$	0.8	0.03	3

**P-value** $Tbx2^{cre/+}$ vs. $Tbx2^{cre/cre};Cdkn1a^{-/-}$; $Cdkn1b^{-/-}$	0.0035
**P-value** $Tbx2^{cre/cre}$ vs. $Tbx2^{cre/cre};Cdkn1a^{-/-}$; $Cdkn1b^{-/-}$	0.0016

statistics of RT-PCRs of E18.5 lungs of compound mutants of *Tbx2, Cdkn1a* and *Cdkn1b* referring to figure 6C

	Cdkn1a	standard deviation	*Cdkn1b*	standard deviation
$Tbx2^{cre/+}$	1.000	0.718	1.000	0.705
$Cdkn1a^{-/-}$	0.008	0.308	1.974	0.676
$Cdkn1b^{-/-}$	2.096	0.540	0.172	0.145
$Tbx2^{cre/cre}$	1.568	1.027	3.136	0.234
$Tbx2^{cre/cre};Cdkn1a^{-/-}$	0.338	0.321	2.821	0.364
$Tbx2^{cre/cre};Cdkn1b^{-/-}$	9.423	4.213	0.285	0.126

E

BrdU labeling index of $Tbx2^{cre/+};Hprt^{TBX2/+}$ E18.5 mice referring to figure 7B

E18.5	average	standard deviation	p-value wt vs $Tbx2^{cre/cre}$	n
wildtype	5.5%	0.7%		3
$Tbx2^{cre/+};Hprt^{TBX2/+}$	13.5%	0.5%	<0.0005	3

statistics of RT-PCRs of $Tbx2^{cre/+};Hprt^{TBX2/+}$ E18.5 lungs referring to figure 7C

	Bmp2	*Bmp4*	*Bmpr2*	*Bmpr1a*	*Msx1*	*Fgf10*	*Fgfr2*	*Etv4*	*Shh*	*Ptchd1*	*Wnt2*	*Wnt5a*	*Axin2*
$Tbx2^{cre/+};Hprt^{TBX2/+}$	0.56	0.91	2.32	1.10	0.90	1.08	0.81	0.88	1.04	0.85	not expressed	not expressed	1.14
standard deviation	0.28	0.30	0.18	0.33	0.28	0.38	0.42	0.28	0.25	0.33	not expressed	not expressed	0.36
p-value	ns	ns	0.044	ns	ns	ns	ns	ns	sn	ns			ns

statistics of RT-PCRs of $Tbx2^{cre/+};Hprt^{TBX2/+}$ E18.5 lungs referring to figure 7C

	Ccnd1	*Ccnd2*	*Ccnd3*	*Cdk1*	*Cdkn2a_1*	*Cdkn2a_2*	*Cdkn1a*	*Cdkn1b*	*Cdkn1c*	*Tbx3*
$Tbx2^{cre/+};Hprt^{TBX2/+}$	1.29	0.95	1.12	not expressed	not expressed	not expressed	0.22	0.28	not expressed	not expressed
standard deviation	0.36	0.16	0.23				0.08	0.12		
p-value	ns	ns	ns				<0.0005	<0.0005	ns	ns

F

relative organ weight of $Tbx2^{cre/+};Hprt^{TBX2/+}$ P40 mice referring to figure 8B

	relative lung weight (wt set to 1)	standard deviation	p-value wt vs. $Tbx2^{+/-}$, $Hprt^{TBX2/+}$	n
wt	1.000	0.063		3
$Tbx2^{cre/+};Hprt^{TBX2/+}$	1.268	0.024	0.009	3

	relative spleen weight (wt set to 1)	standard deviation	p-value wt vs. $Tbx2^{+/-}$, $Hprt^{TBX2/+}$	n
wt	1.000	0.075		3
$Tbx2^{cre/+};Hprt^{TBX2/+}$	1.089	0.082	0.175	3

statistics of BrdU labeling index of $Tbx2^{cre/+};Hprt^{TBX2/+}$P40 mice referring to figure 8D

P40	average	standard deviation	p-value wt vs. $Tbx2^{cre/cre}$	n
wildtype	1.8%	0.8%		3
$Tbx2^{cre/+};Hprt^{TBX2/+}$	30.6%	4.8%	0.0005	3

G

number of branching endoints referring to figure S1B

	Start	standard deviation	n	p-value
wt	17.0	0.7	7.0	nd
$Tbx2^{-/-}$	17.0	1.0	3.0	

	2d of culture	standard deviation	n	p-value
wt	38.3	3.9	7.0	0.5
$Tbx2^{-/-}$	40.3	1.5	3.0	

	4d of culture	standard deviation	n	p-value
wt	79.0	13.2	6.0	0.08
$Tbx2^{-/-}$	65.0	1.0	3.0	

	6d of culture	standard deviation	n	p-value
wt	116.0	8.7	5.0	1.3E-4
$Tbx2^{-/-}$	70.3	2.1	3.0	

H

number of branching endoints referring to figure S8B

	Start	standard deviation	n	p-value
wt	17.0	0.7	7.0	nd
$Tbx2^{cre/+};Hprt^{TBX2/+}$	15.0	1.4	3.0	

	2d of culture	standard deviation	n	p-value
wt	38.3	3.9	7.0	0.5
$Tbx2^{cre/+};Hprt^{TBX2/+}$	35.0	4.2	3.0	

	4d of culture	standard deviation	n	p-value
wt	79.0	13.2	6.0	0.7
$Tbx2^{cre/+};Hprt^{TBX2/+}$	82.5	2.1	3.0	

	6d of culture	standard deviation	n	p-value
wt	116.0	8.7	5.0	0.6
$Tbx2^{cre/+};Hprt^{TBX2/+}$	111.5	14.8	3.0	

I

relative organ weight of wt, $Cdkn1a^{-/-};Cdkn1b^{-/-}$ and $Cdkn1a^{-/-};Cdkn1b^{+/-}$ P40 mice referring to figure S10B

	relative body weight (wt set to 1)	standard deviation	p-value vs. wt	n
wt	1.00	0.04		3
Cdkn1a-/-;Cdkn1b-/-	1.17	0.01	nd	2
Cdkn1a-/-;Cdkn1b+/-	1.19	0.05	nd	2

	relative lung weight (wt set to 1)	standard deviation	p-value vs. wt	n
wt	1.00	0.04		
Cdkn1a-/-;Cdkn1b-/-	1.35	0.03	nd	2
Cdkn1a-/-;Cdkn1b+/-	1.08	0.06	nd	2

statistics of BrdU labeling index of $Cdkn1a^{-/-}$, $Cdkn1b^{-/-}$ P40 mice referring to figure S10D

P40	average	standard	p-value wt vs.	n
wildtype	1.7%	0.8%		3
$Tbx2^{cre/+};Hprt^{TBX2/+}$	7.4%	1.2%	nd	2
