# Supplementary material for: Tbx2 Controls Lung Growth by Direct Repression of the Cell Cycle Inhibitor Genes Cdkn1a and Cdkn1b
Source: PLoS Genet. 2013 Jan 17;9(1):e1003189. doi: 10.1371/journal.pgen.1003189 (PMC3547831; doi:10.1371/journal.pgen.1003189)
Supplement: Table S2 — Primers and conditions of PCRs for genotyping of mouse strains. (PDF) [file pgen.1003189.s013.pdf]

Tbx2cre			
TBX2cre reverse	GCT AGA GCC TGT TTT GCA CGT TCA	95°C	10 min
wildtype sense	AAGCGTAAAATCAGCAATCAAGG	95°C	1 min] - 5x
wildtype reverse	AAGGCGGACATGGGGAAGTCG	58°C	1 min]
wildtype 450bp		72°C	2 min]
mutant 900bp		95°C	1 min] - 32x
		55°C	1 min]
		72°C	2 min]
		72°C - 8 min	

Hprt		94°C – 4min	
HPRT locus sense (common Primer)	AGGGTCTATTTCTCCTAAGG	94°C – 45sec	--------
CAG promoter antisense (mutant)	CGTCATTGACGTCAATAGGG	54°C – 45 sec	30 cycles
HPRT locus antisense (wildtype)	GCAGTGAGGTAAGCCCAACGC	72°C – 1:45 mi	--------
wildtype 547bp		72°C – 15min	
mutant 756bp		8°C – HOLD	

Cdkn1a			
p21-exon-144	GAACTTTGACTTCGTCACGG	95°C - 5 min	
p21-genoU	ACAACACCTCCTGGTCAGAGG	--------	
p21 PGK-neo3	GAAGAACGAGATCAGCAG	95°C - 45 sec	--------
wildtype 685bp		56°C - 45 sec	40 cycles
mutant 150bp		72°C - 45 sec	--------

		72°C	5 min ---
		8°C	HOLD

Cdkn1b		95°C - 5 min	
P27 KO NEO1	CCTTCTATCGCCTTCTTGACG	--------	
P27 KO MGK3	TGGAACCCTGTGCCATCTCTAT	95°C - 30 sec	-------
P27 wt K5	GAGCAGACGCCCAAGAAGC	58°C - 30 sec	40 cycles
wildtype 1000bp		72°C - 60 sec	-------
mutant 500bp		--------	
		72°C	5 min ---
		8°C	HOLD
