# Supplementary material for: Tbx2 Controls Lung Growth by Direct Repression of the Cell Cycle Inhibitor Genes Cdkn1a and Cdkn1b
Source: PLoS Genet. 2013 Jan 17;9(1):e1003189. doi: 10.1371/journal.pgen.1003189 (PMC3547831; doi:10.1371/journal.pgen.1003189)
Supplement: Table S3 — Primers and conditions for analysis of expression by semi-quantitative RT-PCR. (PDF) [file pgen.1003189.s014.pdf]

gene		sequence	size	annealing temperature °C	annealing time	elongation time
Bmp2	forward	TTTTCCCACTCATCTCTGGAA	100bp	58	30sec	30sec
	reverse	AGACCACCGGCTGGAGAG				
Bmp4	forward	ACTGCCGCAGCTTCTCTGAG	450bp	50	30sec	30sec
	reverse	TTCTCCAGATGTTCTTCGTG				
BmpR2	forward	ACAGAATGTTGACAGGAGACCG	650bp	60	30sec	45sec
	reverse	CTCCTTCTAGCACTTCTGGTGC				
(Bmpr1a) Alk3	forward	GGCAATGACTTTCACCTGCT	100bp	58	30sec	30sec
	reverse	GGTCAAAGCTGTTCGGAGAA				
Msx1	forward	CCTCAAGCTGCCAGAAGATG	175bp	56	30sec	30sec
	reverse	AGCTGAGCTGTGGTGAAAGG				
Fgf10	forward	TGGCTTTGACGGCAACAACTCC	120bp	56	30sec	30sec
	reverse	CGATTGAGAAGAACGGCAAGG				
Fgfr2	forward	GCTGCTACCCAAGGAATCGTTC	120bp	56	30sec	30sec
	reverse	GCTGTTGCTGTTGTTACTGCTGTTC				
Etv4 (Pea3)	forward	ATCATGCAGAAGGTGGCTGG	100bp	56	30sec	30sec
	reverse	CCGGTCAAACTCAGCCTTCA				
Shh	forward	CTGGCCAGATGTTTTCTGGT	565bp	56	30sec	30sec
	reverse	CCACGGAGTTCTCTGCTTTC				
Ptc1	forward	CTGCGGCAAGTTTTTGGTTG	161bp	60	30sec	30sec
	reverse	CCTCTTCTCCTATCTTCTGACGGG				
Wnt2	forward	AGAGGAAAGGCAAGGATGCC	161bp	58	30sec	30sec
	reverse	TTGCATGTGTGCACGTCCAG				
Wnt5a	forward	CACAATCACGCCCACATTTG	491bp	56	30sec	30sec
	reverse	GCCATTAGGAAGAACTTGGAAGAC				
Axin2	forward	CAGCCCTTGTGGTTCAAGCT	101bp	60	30sec	30sec
	reverse	GGTAGATTCCTGATGGCCGTAGT				
CyclinD1	forward	ATGGAACACCAGCTCCTG	850bp	56	30sec	30sec
	reverse	CCAGACCAGCCTCTTCC				
CyclinD2	forward	TCTCTCTCTCCCTCTCTCTCTTTGC	597bp	60	30sec	30sec
	reverse	TTACCCAACACTACCAGTTCCCAC				
CyclinD3	forward	CACGCCCCTGACTATTGAGAAG	244bp	60	30sec	30sec
	reverse	CATCGCAAAGGTGTAATCTGTAGC				
Cdk1	forward	GAAGATCAGACTTGAAAGCG	770bp	56	30sec	30sec
	reverse	CCAAGTCATCAAAGTACGGG				
Cdkn2a_1 (p16)	forward	GGGATCCGCTGCAGACAGACTGGCCAG	516bp	55	30sec	60sec
Cdkn2a_1 (p16) and Cdkn2a_2 (p19Arf)	reverse	TGAGGCCGGATTTAGCTCTG	586bp	55	30sec	60sec
Cdkn2a_2 (p19Arf)	forward	CTTGGTCACTGTGAGGATTC		55	30sec	60sec
Cdkn1a	forward	GTACTTCCTCTGCCCTGCTG	787bp	55	30sec	60sec
	reverse	CACAGAGTGAGGGCTAAGGC				
Cdkn1b	forward	AAACGTGAGAGTGTCTAACG	525bp	55	30sec	60sec
	reverse	CCGTCTGAAACATTTTCTT				
Cdkn1c	forward	GGACCTTTCGTTCATGTAGC	108bp	55	30sec	60sec
	reverse	ACATGGTACAGAGTGTTCTCA				
Tbx3	forward	CCAGGCTGCGTTACAGCC	350bp	56	30sec	30sec
	reverse	CAAACTGGAATGGAGAGACC				
